# Forecasting of energy consumption by G20 countries using an adjacent accumulation grey model

**DOI:** 10.1038/s41598-022-17505-4

**Published:** 2022-08-04

**Authors:** Ijlal Raheem, Nabisab Mujawar Mubarak, Rama Rao Karri, T. Manoj, Sobhy M. Ibrahim, Shaukat Ali Mazari, Sabzoi Nizamuddin

**Affiliations:** 1 Department of Chemical Engineering, Faculty of Engineering and Science, Curtin University, 98009 Miri Sarawak, Malaysia; 2 Petroleum and Chemical Engineering, Faculty of Engineering, Universiti Teknologi Brunei, Bandar Seri Begawan, 1410 Brunei Darussalam; 3 Department of Physics and Materials Science and Engineering, Jaypee Institute of Information Technology, Noida, 201309 India; 4 Department of Biochemistry, College of Science, King Saud University, P.O. Box: 2455, Riyadh, 11451 Saudi Arabia; 5 Department of Chemical Engineering, Dawood University of Engineering and Technology, Karachi, 74800 Pakistan; 6 School of Engineering, RMIT University, Melbourne, 3000 Australia

**Keywords:** Environmental sciences, Environmental social sciences, Energy science and technology, Engineering

## Abstract

This paper studies an adjacent accumulation discrete grey model to improve the prediction of the grey model and enhance the utilization of new data. The impact of COVID-19 on the global economy is also discussed. Two cases are discussed to prove the stability of the adjacent accumulation discrete grey model, which helped the studied model attain higher forecasting accuracy. Using the adjacent accumulation discrete grey model, non-renewable energy consumption in G20 countries from 2022 to 2026 is predicted based on their consumption data from 2011 to 2021. It is proven that the adjacent accumulation exhibits sufficient accuracy and precision. Forecasting results obtained in this paper show that energy consumption of all the non-renewable sources other than coal has an increasing trend during the forecasting period, with the USA, Russia, and China being the biggest consumers. Natural gas is the most consumed non-renewable energy source between 2022 and 2026, whereas hydroelectricity is the least consumed. The USA is the biggest consumer of Nuclear energy among the G20 countries, whereas Argentina consumed only 0.1 Exajoules of nuclear energy, placing it at the end of nuclear energy consumers.

## Introduction

Global industrialization and urbanization have entered a crucial stage and are expected to play a role in global economic growth in the next 10 or 20 years. However, countries must consume high energy for rapid urbanization and industrialization to reach their financial targets^1^. Due to global warming threats and limited resources, it is not sustainable to keep consuming natural resources in high amounts in the long run. It is significant to control and reduce energy consumption growth globally, which is impossible until accurate energy consumption data of developed countries^2^.

Forecasting is a technique that uses small data to extract valuable insight and can be used for both short-term and long-term forecasts^3^. Energy consumption forecasting is a technique many countries use to predict the future prosperity of the energy consumption pattern^4^. Governments and businesses use energy consumption forecasting to determine their policies and strategies related to energy consumption for the upcoming years^5^.

Grey models are widely used to forecast energy consumption by many countries. Previously, a grey prediction model with a very grey average weakening buffer operator was used to forecast China’s shale gas output. Apart from that, a new fractional accumulation grey model was used to forecast the nuclear energy consumption of China.

Many forecast models are available, but the grey system theory, which Deng Julong proposed in the 1980s, has gained the attention of many researchers and scholars. Scholars have considered different aspects to improve the grey model^4^. For instance, the model proposed by Markov optimizes the background value and improves the central point weight function^6^. Bernoulli’s model proposed the nonlinear grey model that optimizes the initial condition with boundary value^7^. Nevertheless, the above-given models are only suitable for minor scale problems. Therefore, an optimized grey system theory was proposed for more big sample data with adjacent accumulation DAGM (1,1)^8^.

During the construction step, the grey model treats every model equally. Still, since the significance of each sample that contributes to the construction of the prediction model cannot be the same, it is feasible to estimate the weight of each piece independently to obtain better results^9^. In the discrete adjacent grey model, the sample size does not change or affect the forecasting model, making the percentage errorless and more stable^10^.

The discrete grey model has been previously used to predict the energy consumption of the Asia–Pacific Economic Cooperation^10^. Considering the size and magnitude of the G20, it is significant to predict their non-renewable energy consumption. G20 was formed in 1999; it consists of World’s largest economies. Every year, G20 discusses financial stability and international economic by bringing together the most important developing and industrialized economies^11^.

**Figure 1:**
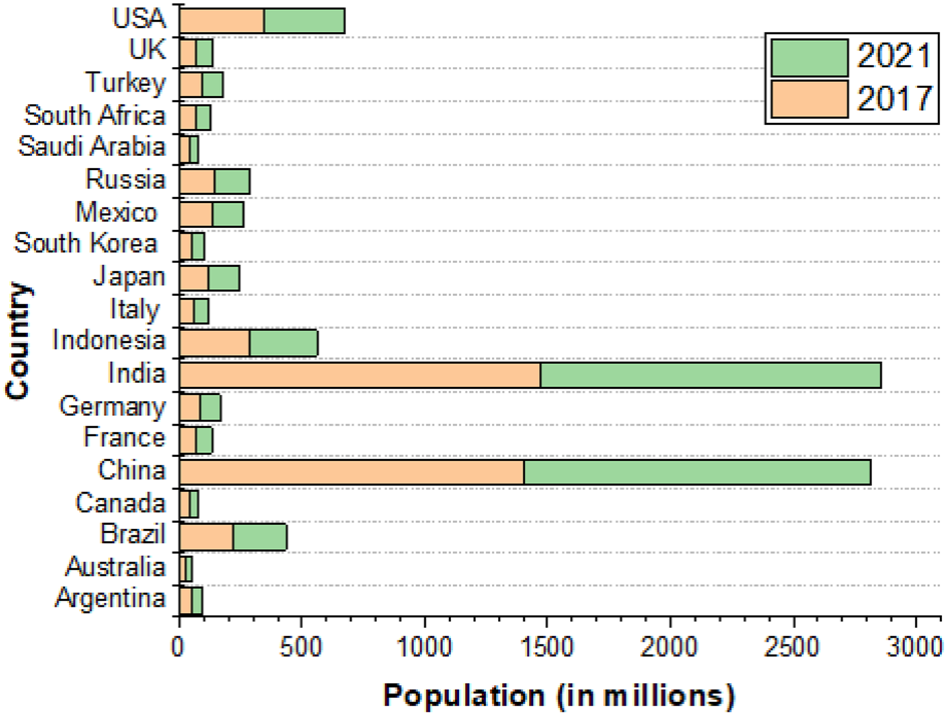
Population growth in G20 countries between 2021 and 2027 (in millions)^13^.

G20 represents 80% of global trade, 60% of world’s population and 85% of global GDP. Its objective is to create financial mechanism, risk reduction, sustainable growth, energy policy and coordinate policy between its member countries^12^. Figure 1 shows the contribution of G20 in world population in millions. So, considering their non-renewable energy consumption, which results in an increase in carbon dioxide emission and global warming and is highly predicted to cause energy and fossil fuel shortfall in the future, it is critical to forecasting the energy consumption of G20 countries for the next 5 years. So, the relevant authorities can architect their energy policy for the future.

In this paper, non-renewable energy consumption in G20 countries is predicted by using DAGM (1,1). Results are obtained for non-renewable energy consumption prediction in G20 countries. Hence, this paper makes the following contributions.

- The background of the grey model is studied, and its comparison is reviewed to analyze the performance and accuracy of other grey models and DAGM (1,1).
- DAGM (1,1) weakens the constraints of GM (1,1) as it can be applied to exponential growth data and improves the development of the grey forecasting model.
- The relationship between DAGM (1,1) and other forecasting grey models is also studied to estimate the accuracy of DAGM (1,1). The accuracy of model and coefficient development was analyzed according to actual data.
- The model's validity was analyzed by studying the non-renewable energy consumption in G20 countries. The obtained results show that the new model can be applied to other energy consumption forecasting and that it has predicted the non-renewable energy consumption in G20 countries to propose effective policy recommendations to the corresponding countries.

## Literature review

A Series of ecological and environmental issues, such as extreme weather, greenhouse effects and global warming, affect human survival and development. High carbon emission issues have resulted from increased non-renewable energy consumption and rapid urbanization. Energy demand is expected to grow globally due to economic and population growth^14^. Short-term pollutants and greenhouse gases are mainly emitted from fossil fuel combustion. Human activities, especially GHG emissions, cause climate change in this industrial age. Energy consumption releases approximately 35 billion tons of carbon dioxide into the atmosphere each year. Therefore, it is significant to develop energy consumption plans by developed countries to reduce global carbon emissions^15^.

Annually, due to ongoing urbanizing, a huge number of people have been moving from rural areas to urban areas worldwide. Over the past four decades, the urban population has increased from 39.1% in 1980 to 56.61 in 2021 worldwide^16^. Nevertheless, it is generally acceptable that economic development and quality of life can be improved by urbanization but it also causes other challenges such as energy demand and energy consumption by stimulating which makes urbanization another factor of staggering increase in CO_2_ emission level over the last four decades^17^. Relationship between energy consumption and economic factors in few developed countries are tabulated in Table 1.

**Table 1:** Relationship between energy consumption and economic factors.

Study region	Method	Variables	Main findings	References
China and Taiwan	Engle–Granger	Energy consumption: coal, natural gas, electricity, GDP	GDP → OC	^18^
India	ARDL bounds testing approach	GDP, high speed diesel	HSD ↔ GDP GDP ↔ HDC	^19^
Portugal	ARDL bounds test	Primary energy consumption, GDP	OC ↔ GDP	^20^
China	Granger causality tests	GDP, OC	OC → GDP	^21^
South Korea	Granger causality tests	GDP, OC	OC → GDP	^22^

### Impact of COVID-19

Global economy and energy sector were majorly affected by COVID-19 pandemic. For a affect, it had caused worst damage than World War II as the global economy was shocked by it^23^. Uncertainties about long-lasting economic crisis were justified by the safety measures such as travel restrictions, border shutdowns and quarantine to make the pandemic curve linear^24^. Demand for many services and good were significantly declined by induced economic loss due to COVID-19. Consequently, demand for fossil fuel was drastically hindered all over the world^25^. According to IEA global energy review, energy demand was declined by 3.8% from January to March 2020 and further decline by 6% at the end of 2020^26^. Decline in energy consumption from January 2020 to March 2020 also resulted in reduction of carbon dioxide emission in 2020^27^.

**Figure 2:**
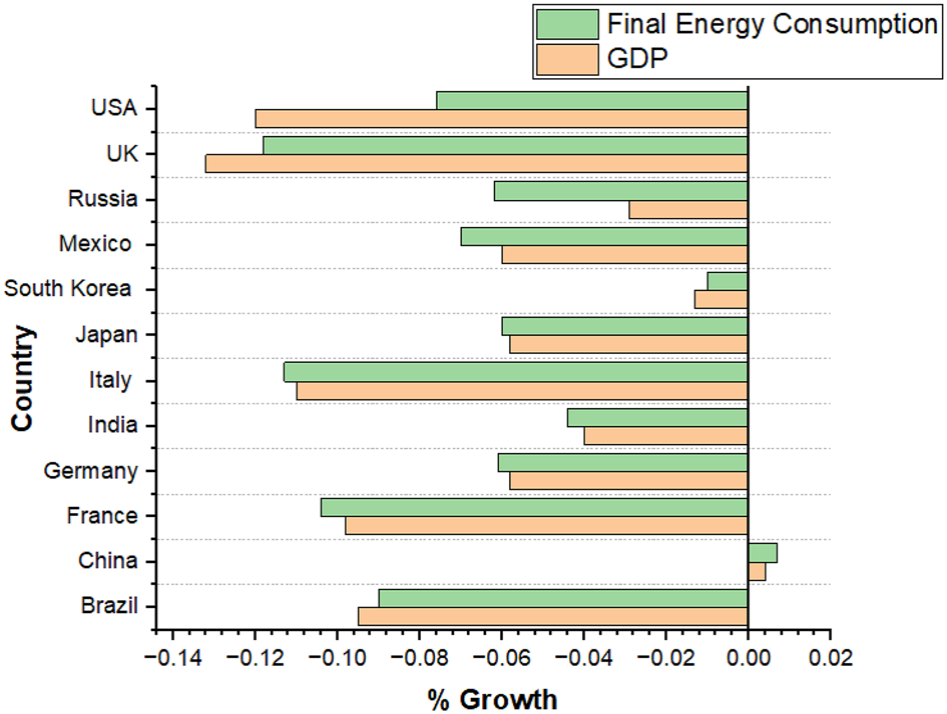
Relationship between energy consumption and GDP in 2020^28^.

Additionally, economy dependent on non-renewable energy like oil, gas and coal was significantly decreased by 5%, 2% and 8%, respectively. It is predicted by the researchers that global energy economic crisis by the end 2030 can be greater than the energy economic crisis in 2008^26^. Figure 2 shows the relationship Between Energy Consumption and GDP growth in 2020.

### Significance of forecasting

Forecasting is defined as a predicting future trend by taking into consideration past and present trends. Energy forecasting is very crucial as it can help global stakeholders to plan and implement their energy policies. G20 countries are major energy consumers in the World due to their rapid industrialization and economic growth. Therefore, accurate forecast of energy consumption can help these countries to assess the future energy demand and supply situation in their countries by making readjustments and optimization in energy structure, and diversify energy use. Based on forecasting and optimization, allocation of social resource and healthy growth of economy can be focused.

### Forecasting methods

Most commonly used prediction methods are given below.

#### Intelligent prediction methods and statistical method

A hybrid intelligent approach was developed by applying an adaptive algorithm to evaluate energy consumption, GDP, import and export data and taking population as inputs^29^. Artificial neutral network ARX (ANNARX) and conventional autoregressive exogenous (ARX) model were used to predict Malaysian oil data in 2020 on the basis of its per capita GDP, population and oil consumption data. Oil consumption in Canada, Japan and Germany were predicted by applying and adaptive neuro-fuzzy inference system which was optimized with a sine–cosine algorithm^30^. Apart from that, fuel consumption by marine engines were predicted by different machine learning models^31^. Malaysia’s oil demand was predicted by applying regression model whereas long term correlation between energy consumption and economic factors, labor productivity, oil prices and other influencing factors were predicted by using cointegration method^32^.

#### Grey model

Various uncertainty theories are emerged due to limited and uncertain information obtained by people. For instance, Fuzzy mathematics and Rough sets theories were proposed but system structure could realize only small part due to the limited knowledge and information^33^. To resolve this issue, grey system theory was proposed by Deng in 1982^34^. Many fields have successfully applied grey system theory. There are five main categories of grey prediction such as:

- Time series forecasting.
- Calamity forecasting.
- Seasonal calamity forecasting.
- Topological forecasting.
- Systematic forecasting.

GM (1,1) is main model of grey theory of prediction, i.e., single variable first order grey model which can provide high precision results by creating with few data (four or more). GM (!,1) model is one of the most used method in grey system^35^. Recently, it has exhibited satisfactory results after getting applied in many fields. Despite implementation of GM (1,1) model in many fields its performance can still be improved^36^. Many techniques to optimize the precision of model have been proposed over the years. Residual sequence amendable method was provided by Deng^10^. Method for optimum grey derivative’s whitening values were provided by Mu in which he proposed a method to estimate parameters by developing an unbiased GM (1,1). Model GM (1,1) was amended by providing the center approach in which adjusting grey model was derived^37^. The background values of structure method were provided in model GM (1,1), which showed strong adaptability by reestablishing a simple calculating formula^38^. Optimum time response sequence was provided by utilizing the least square method in order to find the constant number c in basic equation of GM (1,1), eventually optimum time response sequence was created for model GM (1,1)^39,40^. Forecasting precision can be advanced by all these methods, apart from that it also reduces error partially but problem could not be solved completely.

Many studies have been conducted to improve the application of Grey model, traditional GM (!,1) and other modelling technology are integrated in these improved Grey models but it does not include research Grey model’s accumulated generating operator^7^. Rolling mechanism was used to emphasize the principle of new information of small sample in Grey model. Rolling mechanism assisted Grey model in keeping equal series dimensions upon removing the older data and adding new one^41^.

Once the results are obtained, new data is added at the end of series after older data is deleted from system. It is recommended to use rolling mechanism Grey model for recent data, so the accuracy of forecasting could be increased for future prediction^42^. The future law of development for system with small data is represented by the new data. Hence, significance and principle of new information in grey model theory was put forward by researchers. To consider the principle of new information priority, a novel Grey model with fractional accumulation is used in this paper which indicates that used GM (1,1) has higher performance and precision not only for forecasting but also for model fitting^43^.

### Performance evaluation of grey model

To analyze the accuracy and performance of studied discrete grey model, it is compared with other forecasting models by discussing two different cases.

#### Case 1

In this case, four models were used to predict the added value of the tertiary industry in China using the data from 2005 to 2012 to forecast 2013 to 2014. The predicted values of the model are shown in Table 2. It can be seen that DAGM (1,1) possesses smaller MAPE values as compared to the other four models irrespective of the error of fitting result; hence it has been proven that DAGM (1,1) is very much effective^44^. Table 2 shows the forecasting results of different forecasting grey models.

**Table 2:** Case 1: Forecasting results of different grey models^44^.

Year	Actual value	GM (1,1)	RPE %	EOGM (1,1)	RPE %	DAGM (1,1)^10^	RPE %
2005	1590.7	1590.7	0	1590.7	0	1590.7	0
2006	1815.3	1896.7	4.484107	1847.3	1.762794	1825.6	0.567399
2007	2106.7	2084.3	1.06327	2077.8	1.37181	2073.4	1.58067
2008	2327.1	2291.1	1.54699	2302.6	1.05281	2320.5	0.28361
2009	2547.4	2518.5	1.13449	2536.2	0.43966	2568.1	0.812593
2010	2794.1	2768.5	0.91622	2786.9	0.25769	2813.1	0.680004
2011	3059.7	3043.2	0.53927	3056.4	0.10785	3058.5	0.03922
2012	3303.3	3345.3	1.271456	3333.8	0.923319	3303.3	0
MAPE	–	38.65	–	21.15	–	15.85	–
MAE	–	31	–	17.2	–	11.26	–
RMSE	–	1.37	–	0.74	–	0.49	–
2013	3576	36,773	2.83277	3650.3	2.07774	3547.6	0.79418
2014	3856.6	4042.3	4.815122	3987.4	3.391588	3791.3	1.6932
MAPE	–	149.58	–	106.37	–	50.35	–
MAE	–	143.5	–	102.55	–	46.85	–
RMSE	–	3.82	–	2.73	–	1.24	–

#### Case 2

This model was also applied in Jiangsu province as the conventional GM (1,1) only satisfies the forecasting for small samples. Generally, Higher predictive accuracy can be obtained when the sample volume is small during the forecasting process. Hence, the accuracy of DAGM (1,1) is proven by applying it to smaller volume samples and then comparing it to GM (1,1). It is shown in Table that DAGM (1,1) possesses better results as it has a lesser influence on disturbance boundary as compared to GM (1,1)^45^. Table 3 shows the comparison of discrete grey model with other forecasting results.

**Table 3:** Case 2: Forecasting results of different grey models^45^.

Year	Actual value	GM_6_(1,1)	RPE %	GM_4_(1,1)	RPE %	DAGM (1,1)^10^	RPE %
2000	132.4	132.4	0	–	–	132.4	0
2001	144.6	142	1.79806	–	–	142.3	1.59059
2002	156.3	157.3	0.639795	156.3	0	155.8	0.3199
2003	1737	174.2	0.287853	172.1	0.92113	172.2	0.86356
2004	190.2	193	1.472135	192.5	1.209253	192.1	0.998948
2005	216.7	213.8	1.33826	215.3	0.64605	216.2	0.23073
RMSE	–	1.12	–	0.93	–	0.66	–
MAE	–	1.63	–	1.33	–	1.12	–
MAPE	–	2.01	–	1.57	–	1.39	–
2006	249.4	236.8	5.05213	240.8	3.44828	245.4	1.60385
RMSE	–	5.04	–	3.46	–	1.61	–
MAE	–	12.6	–	8.6	–	4	–
MAPE	–	12.6	–	8.6	–	4	–

## Methodology

Grey Model^46^

$${y}^{\left(0\right)}=\left({y}^{\left(0\right)}\left(1\right),{y}^{\left(0\right)}\left(2\right),{y}^{\left(0\right)}\left(3\right),{y}^{\left(0\right)}\left(4\right)\dots {y}^{\left(0\right)}\left(n\right)\right),$$

where n represents the total number of periods and is greater or equal to 5.

The precision of GM (1,1) can be increased by deriving the accumulated generating operator through original time series data. The accumulated generating data operator is derived.

$${y}^{\left(1\right)}\left(k\right)=\sum_{m=1}^{n}{y}^{\left(0\right)}\left(m\right),k=\mathrm{2,3}\dots, n.$$

Obtained time series data can then be expressed as,

$${y}^{\left(1\right)}=\left({y}^{\left(1\right)}\left(1\right),\sum_{m=1}^{2}{y}^{\left(0\right)}\left(m\right),\sum_{m=1}^{3}{y}^{\left(0\right)}\left(m\right),\sum_{m=1}^{4}{y}^{\left(0\right)}\left(m\right),\dots \sum_{m=1}^{n}{y}^{\left(0\right)}\left(m\right)\right),$$

$${y}^{\left(1\right)}\left(1\right), {y}^{\left(1\right)}\left(2\right),{y}^{\left(1\right)}\left(3\right)\dots \dots \dots {y}^{\left(0\right)}\left(n\right)).$$

**Figure 3:**
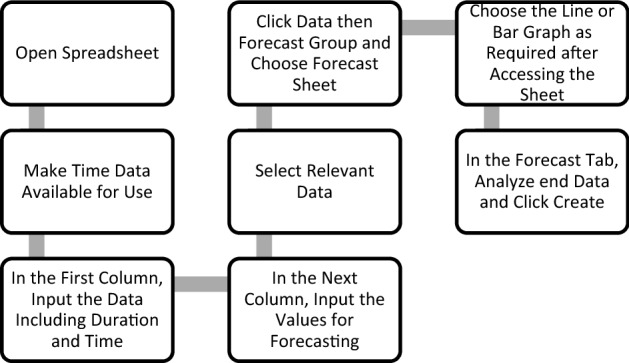
Flowchart to estimate outcomes by time series model.

Given below Fig. 3 shows the steps to follow to obtain time series model.

Once the forecasting model is setup, formulate best estimation of future consumption by moving onto interpretation.

Grey differential equation is then used to construct the GM (1,1) model.

$${y}^{\left(0\right)}\left(k\right)+ {az}^{\left(1\right)}\left(k\right),=u,$$

where a = Development coefficient, u = Grey Input, z = Background value.

$${z}^{\left(1\right)}\left(k\right)=\frac{1}{2}\times \left[{y}^{\left(1\right)}\left(k\right)+{y}^{\left(1\right)}\left(k-1\right)\right], k=2, 3\dots .n,$$

where

$${y}^{\left(1\right)}\left(k\right)={y}^{\left(0\right)}\left(`\right)-\frac{u}{a}\times {e}^{-a\left(k-1\right)}+\frac{u}{a},$$

where

$${y}^{\left(1\right)}\left(k\right)={y}^{\left(0\right)}\left(`\right)-\frac{u}{a}\times {e}^{-a\left(k-1\right)}+\frac{u}{a},$$

$$\left[\begin{array}{c}a\\ u\end{array}\right]={({A}^{T}A{)}^{-1}A}^{T}Y,$$

and

$$A=\begin{array}{ccc}-{z}^{\left(1\right)}& 2& 1\\ -{z}^{\left(1\right)}& 3& 1\\ -{z}^{\left(1\right)}& \left(n\right)& 1\end{array},$$

$$Y={[y}^{\left(0\right)}\left(`2\right),{y}^{\left(0\right)}\left(`2\right)\dots {y}^{\left(0\right)}\left(n\right){]}^{T}.$$

An inverse of accumulation grey model is employed to obtain the values for forecasting value, which gives the following forecasting equation^3^.

$$\begin{aligned} y^{\left( 0 \right)} \left( {n + p} \right) = & y^{\left( 1 \right)} \left( {n + p} \right) - y^{\left( 1 \right)} \left( {n + p - 1} \right), \\ = & (y^{\left( 0 \right)} \left( 1 \right) - \frac{u}{a})\left( {1 - e^{0} } \right)e^{{ - a\left( {n + p - 1} \right)}} , p = 1,2,3. \\ \end{aligned}$$

where $${y}^{\left(0\right)}\left(1\right)$$, $${y}^{\left(0\right)}\left(2\right)$$, $${y}^{\left(0\right)}\left(3\right)$$………$${y}^{\left(0\right)}\left(n\right)$$= GM (1,1).

${y}^{\left(0\right)}\left(n+1\right),{y}^{\left(0\right)}\left(n+2\right),{y}^{\left(0\right)}\left(n+3\right)\dots \dots ..{y}^{\left(0\right)}\left(n+P\right)$ = Forecasting value of the model in the period, p

### Discrete grey model and its stability^10^

#### Definition 1

${X}^{\left(0\right)}=\left\{{x}^{\left(0\right)}\left(1\right),{x}^{\left(0\right)}\left(2\right),{x}^{\left(0\right)}\left(3\right),{x}^{\left(0\right)}\left(4\right)\dots \dots \dots {x}^{\left(0\right)}\left(n\right)\right),$ where ${x}^{\left(0\right)}=$ orignal data, $\lambda =$ adjacent accumulation parameter, ${x}^{\left(1\right)}=$ adjacent accumulation generating sequence, ${L[x}^{\left(0\right)}(k)]=$ perturbation bound of ${L[x}^{\left(0\right)}\left(k\right)$].

$${x}^{\left(0\right)}(1)=({x}^{\left(0\right)}\left(1\right),$$

$${x}^{\left(1\right)}\left(2\right)={\lambda x}^{\left(0\right)}\left(1\right)+{x}^{\left(0\right)}\left(2\right),$$

$${x}^{\left(1\right)}\left(3\right)={\lambda x}^{\left(0\right)}\left(2\right)+{x}^{\left(0\right)}\left(3\right),$$

$${x}^{\left(1\right)}\left(n\right)={\lambda x}^{\left(0\right)}\left(n-1\right)+{x}^{\left(0\right)}\left(n\right),$$

where $\lambda =$ Adjacent accumulation parameter, ${X}^{\left(1\right)}=\left\{{x}^{\left(1\right)}\left(1\right),{x}^{\left(1\right)}\left(2\right),{x}^{\left(1\right)}\left(3\right),{x}^{\left(1\right)}\left(4\right)\dots {x}^{\left(1\right)}\left(n\right)\right)$ = Accumulation generating sequence pf ${X}^{\left(0\right)}.$

$\lambda$ can be adjusted during the generation sequence of old and new data.

Then the least-squares estimation parameter is satisfied by

$${x}^{\left(1\right)}\left(k+1\right)={\beta }_{1}{x}^{\left(1\right)}\left(k\right)+{\beta }_{2},$$

$$\widehat{\beta }={(B}^{T}{B)}^{-1}{B}^{T}Y,$$

$${x}^{\left(1\right)}\left(1\right)={x}^{\left(0\right)}\left(1\right),$$

$$\widehat{x}\left(k+1\right)={\beta }_{1}^{k}{x}^{\left(0\right)}\left(1\right)+\frac{1-{\beta }_{1}^{k}}{1-{\beta }_{2}}.{\beta }_{2}, k=\mathrm{1,2},\mathrm{3,4}\dots \dots n-1,$$

$${\widehat{x}}^{\left(0\right)}\left(k+1\right){\widehat{x}}^{1}\left(k+1\right)-{\lambda \widehat{x}}^{\left(0\right)}\left(k+1\right).$$

It is necessary to use the adaject accumulation method to accumulate the original data ${X}^{\left(0\right)}$. The weight of old and new information is adjusted to introduce the adjacent accumulation parameter.

**Figure 4:**
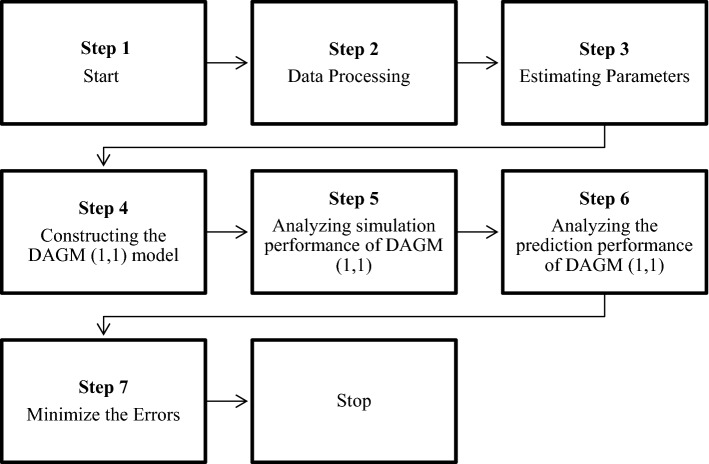
Flow Chart to Design DAGM (1,1)^10^.

To perform the study and simulation, a Discrete adjacent grey model is used. The flowchart of the simulation is shown in Fig. 4. The following lemma then tests the stability of the discrete adjacent grey model.

$$\| \Delta x\| \le \frac{{K}_{\dag}}{{\gamma }_{\dag}}\biggl(\frac{\| \Delta B{\| }_{2}}{\Vert B\Vert }\Vert x\Vert +\frac{\Vert \Delta Y\Vert }{\Vert B\Vert }+\frac{{K}_{\dag}}{{\gamma }_{\dag}}\biggl(\frac{\| \Delta B{\| }_{2}}{\Vert B\Vert }\frac{{K}_{\dag}}{{\gamma }_{\dag}}\left(\frac{\Vert {r}_{x}\Vert }{\Vert B\Vert }\right)=\sqrt{1+{\lambda }^{2}}\left|\varepsilon \right|\frac{{K}_{\dag}}{{\gamma }_{\dag}}\left(\frac{1}{\Vert B\Vert }\Vert x\Vert +\frac{1}{\Vert B\Vert }+\frac{{K}_{\dag}}{{\gamma }_{\dag}}\left(\frac{1}{\Vert B\Vert }\frac{\Vert {r}_{x}\Vert }{\Vert B\Vert }\right)\right).$$

#### Lemma 1

Let $A\in {C}^{m\times n},b\in {C}^{m}$^2^ where ${A}^{t}$= Inverse of matrix A, B = A + E, C = b + $k\in {C}^{m}$, $\| Bx-c{\| }_{2}$ = max, $\| Ax-b{\| }_{2}$ = min.

Then the equation becomes

$$\| h\| \le \frac{{K}_{t}}{{\gamma }_{t}}(\frac{\| E{\| }_{2}}{\Vert A\Vert }\Vert x\Vert +\frac{\Vert k\Vert }{\Vert A\Vert }+\frac{{K}_{t}}{{\gamma }_{t}}\frac{\| E{\| }_{2}}{\Vert A\Vert }\frac{{\| r}_{t}\| }{\Vert A\Vert },$$

where $${K}_{t}$$=$$\Vert A{\| }_{2}^{*}\Vert A\| \cdot {\gamma }_{*}=1-\Vert A{\| }_{2}^{*}\Vert E{\| }_{2}\cdot {r}_{x}=b-Ax$$.

#### Theorem 1^10^

Assume that ${X}^{\left(0\right)}=\{{x}^{\left(0\right)}\left(1\right),{x}^{\left(0\right)}\left(2\right), {x}^{\left(0\right)}\left(3\right), {x}^{\left(0\right)}\left(4\right)\dots \dots \dots {x}^{\left(0\right)}\left(n\right)$ is the original nonnegative sequence, B and Y are supposed to remain the same as mentioned earlier

$$L[{x}^{\left(0\right)}\left(k\right)=\frac{{K}_{\dag}}{{\gamma }_{\dag}}\left(\frac{\| \Delta B{\| }_{2}}{\Vert B\Vert }\Vert x\Vert +\frac{\Vert \Delta Y\Vert }{\Vert B\Vert }+\frac{{K}_{\dag}}{{\gamma }_{\dag}}\frac{\| \Delta B{\| }_{2}}{\Vert B\Vert }\frac{\Vert {r}_{x}\Vert }{\Vert B\Vert }\right), \mathrm{k}=\mathrm{1,2},\mathrm{3,4}\dots ..\mathrm{n},$$

where $L[{x}^{\left(0\right)}\left(k\right)$ = Perturbation Bound.

${\widehat{x}}^{\left(0\right)}\left(k\right)={x}^{\left(0\right)}\left(k\right)+\in and \| {B}^{\dag}{\| }_{2}\Vert \Delta B{\| }_{2}\Vert <1, L[{x}^{\left(0\right)}\left(k\right)$ Does not change with an increase in sample values. Hence, DAGM (1,1) solution is more suitable and stable.

Proof of $${\widehat{x}}^{\left(0\right)}\left(2\right)={x}^{\left(0\right)}\left(2\right)+\in$$

$$\widehat{\beta }=B+\Delta B=\left[\begin{array}{c}{x}^{\left(1\right)}\left(1\right)\quad 1\\ {x}^{\left(1\right)}\left(2\right)\quad 1\\ {x}^{\left(1\right)}\left(3\right) \quad1\\ {x}^{\left(1\right)}\left(4\right) \quad 1\\ .\\ .\\ .\\ .\\ .{x}^{\left(1\right)}\left(n\right)\quad 1\end{array}\right]+\left[\begin{array}{c}0 \quad0\\ \in \quad0\\ \lambda \quad0\\ \\ .\\ .\\ .\\ .\\ .0 \quad0\end{array}\right],$$

$$\widehat{Y}=Y+\Delta Y=\left[\begin{array}{c}{x}^{\left(1\right)}\left(1\right) \\ {x}^{\left(1\right)}\left(2\right)\\ .\\ .\\ .\\ .\\ .\\ .\\ {x}^{\left(1\right)}\left(n\right) \end{array}\right]+\left[\begin{array}{c}0\\ \in \\ \lambda \in \\ \\ .\\ .\\ .\\ .\\ .0 \end{array}\right].$$

B vector is linearly independent

$$\| \Delta Y{\| }_{2}=\sqrt{\left(1+{\lambda }^{2}\right){\epsilon }^{2}}= \sqrt{\left(1+{\lambda }^{2}\right){\epsilon }^{2}}=\sqrt{1+{\lambda }^{2}}|\in |,\Delta {B}^{T}\Delta B=\left[\begin{array}{cc}0& 0\\ 0& \left(1+{\lambda }^{2}\right){\in }^{2}\end{array}\right].$$

$\| \Delta B{\| }_{2}=\sqrt{{\lambda }_{max}\left({\Delta B}^{T}\Delta B\right)}$ whereas the maximum value for ${\Delta B}^{T}\Delta B=\left(1+{\lambda }^{2}\right){\epsilon }^{2}.$ Thus

$$\| \Delta B{\| }_{2}=\sqrt{\left(1+{\lambda }^{2}\right)\left|\in \right|,}$$

$$L\left[{x}^{\left(0\right)}\left(2\right)\right]=\sqrt{1+{\lambda }^{2}}\left|\varepsilon \right|\frac{{K}_{\dag}}{{\gamma }_{\dag}}\left(\frac{1}{\Vert B\Vert }\Vert x\Vert +\frac{1}{\Vert B\Vert }+\frac{{K}_{\dag}}{{\gamma }_{\dag}}\left(\frac{1}{\Vert B\Vert }\frac{\Vert {r}_{x}\Vert }{\Vert B\Vert }\right)\right).$$

Similarly, it has been proven that ${\widehat{x}}^{\left(0\right)}\left(r\right)={x}^{\left(0\right)}\left(r\right)+\in ,$ So

$$L\left[{x}^{\left(0\right)}\left(r\right)\right]=\sqrt{1+{\lambda }^{2}}\left|\varepsilon \right|\frac{{K}_{\dag}}{{\gamma }_{\dag}}\left(\frac{1}{\Vert B\Vert }\Vert x\Vert +\frac{1}{\Vert B\Vert }+\frac{{K}_{\dag}}{{\gamma }_{\dag}}\left(\frac{1}{\Vert B\Vert }\frac{\Vert {r}_{x}\Vert }{\Vert B\Vert }\right)\right).$$

R = 3,4, …, n−2 When

$${\widehat{x}}^{\left(0\right)}\left(n-1\right)={x}^{\left(0\right)}\left(n-1\right)+\in ,$$

$$L\left[{x}^{\left(0\right)}\left(n-1\right)\right]=\left|\varepsilon \right|\frac{{K}_{\dag}}{{\gamma }_{\dag}}(\frac{1}{\Vert B\Vert }\Vert x\Vert +\frac{\sqrt{1+{\lambda }^{2}}}{\Vert B\Vert }+\frac{{K}_{\dag}}{{\gamma }_{\dag}}\left(\frac{1}{\Vert B\Vert }\frac{\Vert {r}_{x}\Vert }{\Vert B\Vert }\right),$$

when

$${\widehat{x}}^{\left(0\right)}\left(n\right)={x}^{\left(0\right)}\left(n\right)+\in ,$$

$$L\left[{x}^{\left(0\right)}\left(n\right)\right]=\frac{{K}_{\dag}}{{\gamma }_{\dag}}\frac{\left|\in \right|}{\Vert B\Vert }.$$

It is to be noted here that $L\left[{x}^{\left(0\right)}\left(n\right)\right]$ It has no correlation with the sample size; hence it does not vary with the sample size. Usually $L\left[{x}^{\left(0\right)}\left(n\right)\right]$ Increases with an increase in sample size in conventional model GM (1,1) make DAGM (1,1) more stable. Figure 4 shows the steps that were followed in designing DAGM (1,1) whereas tools and softwares used are mentioned in Table 5.

**Table 4:** Tools used for this study.

Softwares	Purpose
MS Excel 2016	To analyze the results and get the values
MATLAB	To verify and analyze the results
Microsoft Excel Solver	To perform the Generalized Reduced Gradient (GRG) nonlinear method for optimization
Origin Lab	To plot the graphs and figures

### Future prediction of natural resources in G20 countries

Natural resources always play a very significant role in developing any country. Demand for these resources is constantly increasing due to the rapid urbanization worldwide. The reserves of these resources are declining due to the rapid increase in consumption and the nonrenewable nature of these resources. The concerned authorities must start looking for alternatives to prevent the depletion of these resources. To perform the research, it was considered to select major industrial countries from all over the world; hence G20 countries are chosen to perform the forecast of these resources in G20 countries by analyzing the historical consumption of non-renewable resources. The recorded data is obtained from the 71th British Petroleum World Energy Statistical Year book 2022.

#### Oil

Analyzing the current situation, the G20 countries include all the world's primary consumers, such as China, USA, and Russia. Studying oil consumption in these countries is significant, so the future supply and demand can be predicted. Apart from that, the prediction can also help governments make future energy policies. Table 5 includes all the primary oil consumers in the world from 2011 to 2021 to predict the oil consumption of G20 countries in the next 5 years.

**Table 5:** Oil consumption data of G20 countries^47^.

Country	2011	2012	2013		2014	2015	2016	2017	2018	2019	2020	2021
Argentina	1.22	1.30	1.38		1.37	1.39	1.35	1.34	1.27	1.17	1.07	1.23
Australia	1.97	2.02	2.08		2.07	2.05	2.06	2.16	2.20	2.18	1.88	1.93
Brazil	4.83	5.00	5.27		5.44	4.94	4.70	4.75	4.51	4.54	4.22	4.46
Canada	4.58	4.64	4.62		4.61	4.63	4.61	4.57	4.73	4.70	4.11	4.17
China	19.41	20.36	21.27		22.11	23.80	24.56	25.86	27.12	28.49	28.74	30.60
France	3.45	3.34	3.29		3.19	3.19	3.17	3.18	3.17	3.14	2.68	2.91
Germany	4.73	4.70	4.80		4.67	4.67	4.76	4.87	4.63	4.66	4.22	4.18
India	6.91	7.33	7.38		7.59	8.20	8.99	9.26	9.68	9.99	9.08	9.41
Indonesia	3.05	3.21	3.11		3.09	2.95	2.84	3.05	3.15	3.06	2.70	2.83
Italy	2.98	2.78	2.54		2.42	2.56	2.54	2.57	2.63	2.55	2.11	2.35
Japan	8.78	9.37	8.96		8.51	8.17	7.93	7.81	7.56	7.32	6.49	6.61
South Korea	4.63	4.80	4.81		4.79	5.02	5.47	5.42	5.40	5.35	5.06	5.39
Mexico	3.97	4.04	3.93		3.75	3.70	3.73	3.59	3.51	3.24	2.47	2.56
Russia	6.20	6.30	6.30		6.60	6.34	6.50	6.48	6.55	6.69	6.34	6.71
Saudi Arabia	6.03	6.34	6.36		7.02	7.29	7.36	7.14	6.90	6.78	6.54	6.59
South Africa	1.10	1.13	1.15		1.13	1.25	1.19	1.19	1.19	1.18	0.96	1.04
Turkey	1.34	1.41	1.51		1.55	1.85	1.98	2.07	2.00	2.01	1.84	1.89
United Kingdom	3.14	3.05	3.00		3.00	3.08	3.18	3.19	3.14	3.06	2.35	2.50
United States of America	34.90	34.10	34.66		34.90	35.61	35.86	36.21	37.08	37.02	32.52	35.33
European Union	24.03	23.05	22.47		22.02	22.38	22.81	23.20	23.25	23.19	20.24	21.32

All consumption in exajoules.

#### Natural gas

Natural gas is one of the most significant non-renewable energy sources. Global influential consumers of natural gas are members of the G20^48^. It is critical to decide on the exploitation and utilization of natural gas carefully. For the reasonable utilization of natural gas, it is essential to know the future demand for natural gas so the policymakers can make the relevant decisions. Table 6 shows the natural gas consumption by the G20 countries from 2011 to 2021, which is used in this study to predict the natural gas consumption for the next five years by G20 countries.

**Table 6:** Natural gas consumption data of G20 countries^47^.

Country	2011	2012	2013	2014	2015	2016	2017	2018	2019	2020	2021
Argentina	1.58	1.64	1.66	1.66	1.68	1.74	1.74	1.75	1.68	1.58	1.65
Australia	1.18	1.19	1.25	1.34	1.40	1.36	1.34	1.32	1.58	1.55	1.42
Brazil	0.99	1.17	1.38	1.46	1.55	1.34	1.35	1.29	1.29	1.13	1.46
Canada	3.62	3.58	3.80	3.95	3.97	3.78	3.96	4.16	4.22	4.08	4.29
China	4.87	5.43	6.19	6.78	7.01	7.54	8.69	10.22	11.10	12.12	13.63
France	1.55	1.60	1.63	1.36	1.47	1.60	1.61	1.54	1.57	1.46	1.55
Germany	2.91	2.92	3.06	2.66	2.77	3.06	3.16	3.09	3.21	3.14	3.26
India	2.17	2.01	1.76	1.75	1.72	1.83	1.93	2.09	2.13	2.18	2.24
Indonesia	1.54	1.55	1.60	1.59	1.65	1.61	1.56	1.60	1.58	1.35	1.33
Italy	2.67	2.57	2.40	2.12	2.32	2.43	2.58	2.49	2.55	2.43	2.61
Japan	4.03	4.44	4.45	4.49	4.27	4.19	4.21	4.17	3.89	3.75	3.73
South Korea	1.74	1.89	1.98	1.80	1.64	1.72	1.79	2.08	2.02	2.07	2.25
Mexico	2.55	2.65	2.80	2.84	2.91	2.99	3.10	3.15	3.17	3.01	3.18
Russia	15.68	15.43	15.30	15.20	14.71	15.14	15.52	16.36	16.00	15.25	17.09
Saudi Arabia	3.16	3.40	3.42	3.50	3.57	3.79	3.93	4.04	4.00	4.07	4.22
South Africa	0.15	0.16	0.15	0.15	0.16	0.13	0.14	0.16	0.15	0.14	0.14
Turkey	1.51	1.56	1.58	1.68	1.65	1.60	1.86	1.70	1.56	1.66	2.06
United Kingdom	2.95	2.77	2.75	2.52	2.59	2.90	2.83	2.83	2.80	2.63	2.77
United States of America	23.70	24.77	25.45	26.00	26.77	26.97	26.64	29.58	30.62	29.95	29.76
European Union	14.01	13.76	13.48	11.93	12.48	13.25	13.87	13.61	14.11	13.69	14.28

All consumption in exajoules.

#### Nuclear energy

Nuclear energy is one of the most dangerous forms of energy as one mistake in handling nuclear power can cause loss of many lives and damage for centuries. Hence, it is a policy by all the United Nations members to reduce the use of nuclear energy. There is no doubt that the development of nuclear energy has a significant effect on coal, oil, and gas. Most G20 countries do not make their nuclear energy consumption data available due to the government policy, so predicted consumption is only available based on available data. Table 7 shows the nuclear energy consumption of G20 countries from 2011 to 2021 to predict the consumption of the next five years.

**Table 7:** Nuclear energy consumption data of G20 countries^47^.

Country	2011	2012	2013	2014	2015	2016	2017	2018	2019	2020	2021
Argentina	0.06	0.06	0.06	0.05	0.07	0.08	0.06	0.06	0.08	0.10	0.10
Brazil	0.15	0.15	0.15	0.14	0.14	0.15	0.14	0.14	0.15	0.13	0.13
Canada	0.89	0.89	0.97	1.00	0.94	0.93	0.92	0.91	0.92	0.88	0.83
China	0.83	0.93	1.05	1.25	1.59	1.97	2.28	2.70	3.18	3.32	3.68
France	4.22	4.03	3.99	4.08	4.07	3.73	3.66	3.77	3.63	3.21	3.43
Germany	1.03	0.94	0.92	0.91	0.85	0.78	0.70	0.69	0.68	0.58	0.62
India	0.31	0.31	0.31	0.32	0.36	0.35	0.34	0.36	0.41	0.40	0.40
Japan	1.55	0.17	0.14	–	0.04	0.16	0.27	0.45	0.60	0.39	0.55
South Korea	1.47	1.42	1.31	1.46	1.53	1.50	1.36	1.22	1.33	1.45	1.43
Mexico	0.10	0.08	0.11	0.09	0.11	0.10	0.10	0.12	0.10	0.10	0.11
Russia	1.65	1.68	1.62	1.69	1.82	1.82	1.87	1.87	1.90	1.96	2.01
South Africa	0.12	0.12	0.13	0.13	0.11	0.14	0.13	0.11	0.12	0.13	0.09
United Kingdom	0.66	0.67	0.66	0.60	0.65	0.66	0.65	0.59	0.51	0.46	0.41
United States of America	7.93	7.67	7.82	7.85	7.81	7.84	7.79	7.76	7.76	7.54	7.40
European Union	7.99	7.69	7.59	7.61	7.32	7.10	6.98	6.97	6.97	6.20	6.62

All consumption in exajoules.

#### Coal

Coal is the most reliable source as far as global energy consumption is considered; hence it is critical to analyze the coal consumption by the G20 countries closely. As the G20 countries include all the major coal consumers globally, it is easy to predict the global coal consumption by analyzing the coal consumption of G20 countries. To perform the forecasting, coal consumption data from 2011 to 2021 is used to indicate the consumption in the next five years by the G20 countries. Table 8 shows the coal consumption by G20 countries from 2011 to 2021.

**Table 8:** Coal consumption data of G20 countries^47^.

Country	2011	2012	2013	2014	2015	2016	2017	2018	2019	2020	2021
Argentina	0.05	0.05	0.05	0.06	0.06	0.04	0.05	0.05	0.03	0.04	0.07
Australia	2.13	2.00	1.89	1.88	1.95	1.94	1.88	1.83	1.75	1.69	1.63
Brazil	0.65	0.64	0.69	0.73	0.74	0.67	0.70	0.69	0.65	0.59	0.71
Canada	0.93	0.88	0.86	0.82	0.82	0.77	0.80	0.65	0.61	0.53	0.48
China	79.71	80.71	82.43	82.48	80.92	80.19	80.56	81.05	81.70	82.38	86.17
France	0.41	0.46	0.48	0.36	0.36	0.35	0.38	0.34	0.27	0.19	0.23
Germany	3.28	3.37	3.47	3.33	3.29	3.20	3.01	2.90	2.25	1.81	2.12
India	12.76	14.02	14.87	16.36	16.55	16.88	17.44	18.58	18.59	17.40	20.09
Indonesia	1.96	2.03	1.78	1.88	2.14	2.23	2.39	2.84	3.41	3.25	3.28
Italy	0.64	0.66	0.57	0.55	0.52	0.46	0.40	0.37	0.28	0.21	0.23
Japan	4.62	4.88	5.07	4.99	5.03	5.02	5.10	4.99	4.91	4.57	4.80
South Korea	3.50	3.38	3.41	3.53	3.58	3.41	3.61	3.63	3.44	3.02	3.04
Mexico	0.62	0.54	0.53	0.53	0.53	0.52	0.64	0.57	0.54	0.24	0.23
Russia	3.94	4.12	3.79	3.67	3.86	3.74	3.51	3.63	3.57	3.29	3.41
South Africa	3.79	3.70	3.70	3.75	3.52	3.78	3.72	3.53	3.76	3.56	3.53
Turkey	1.42	1.53	1.32	1.51	1.45	1.61	1.65	1.71	1.76	1.70	1.74
United Kingdom	1.32	1.63	1.55	1.25	0.97	0.46	0.38	0.32	0.22	0.20	0.21
United States of America	19.70	17.42	18.08	18.04	15.58	14.26	13.87	13.28	11.34	9.20	10.57
European Union	10.75	10.70	10.50	10.00	9.98	9.58	9.25	9.04	7.30	5.97	6.74

All consumption in exajoules.

#### Hydroelectricity

Hydroelectricity is used to harness the power of water in motion, such as the flow of water over a waterfall. There are three types of hydroelectricity in which impoundment is the most common one. Hydroelectricity is not commonly used in most G20 countries; hence, a few countries’ consumption data is unavailable. Table 9 shows hydroelectric energy consumption from 2011 to 2021 to predict the consumption in the next 5 years by the G20 countries.

**Table 9:** Hydroelectricity Consumption Data of G20 Countries^47^.

Country	2011	2012	2013	2014	2015	2016	2017	2018	2019	2020	2021
Argentina	0.31	0.29	0.32	0.32	0.31	0.29	0.30	0.31	0.26	0.22	0.18
Australia	0.18	0.16	0.18	0.13	0.13	0.16	0.12	0.16	0.13	0.14	0.15
Brazil	4.25	4.10	3.84	3.64	3.49	3.67	3.55	3.70	3.78	3.75	3.42
Canada	3.73	3.75	3.84	3.73	3.71	3.71	3.78	3.68	3.62	3.65	3.59
China	6.83	8.52	8.92	10.33	10.80	11.11	11.16	11.42	12.08	12.50	12.25
France	0.44	0.58	0.70	0.61	0.53	0.58	0.47	0.61	0.53	0.58	0.55
Germany	0.18	0.21	0.23	0.19	0.18	0.20	0.19	0.17	0.19	0.17	0.18
India	1.31	1.14	1.29	1.36	1.29	1.24	1.30	1.33	1.54	1.55	1.51
Indonesia	0.12	0.13	0.17	0.15	0.13	0.18	0.18	0.21	0.20	0.23	0.23
Italy	0.46	0.41	0.52	0.57	0.43	0.39	0.33	0.47	0.45	0.43	0.41
Japan	0.82	0.76	0.78	0.80	0.83	0.77	0.76	0.77	0.70	0.73	0.73
South Korea	0.05	0.04	0.04	0.03	0.02	0.03	0.03	0.03	0.03	0.04	0.03
Mexico	0.36	0.31	0.27	0.38	0.30	0.30	0.31	0.31	0.22	0.25	0.33
Russia	1.62	1.61	1.78	1.69	1.63	1.78	1.77	1.82	1.84	2.01	2.02
South Africa	0.03	0.01	0.01	0.02	0.01	0.01	0.02	0.01	0.01	0.01	0.01
Turkey	0.52	0.57	0.58	0.40	0.65	0.65	0.56	0.57	0.84	0.74	0.52
United Kingdom	0.06	0.05	0.05	0.06	0.06	0.05	0.06	0.05	0.06	0.06	0.05
United States of America	3.14	2.70	2.62	2.49	2.39	2.54	2.84	2.76	2.71	2.67	2.43
European Union	3.05	3.26	3.60	3.60	3.24	3.30	2.79	3.26	3.02	3.25	3.24

All consumption in exajoules.

## Results and discussion

### Oil

**Figure 5:**
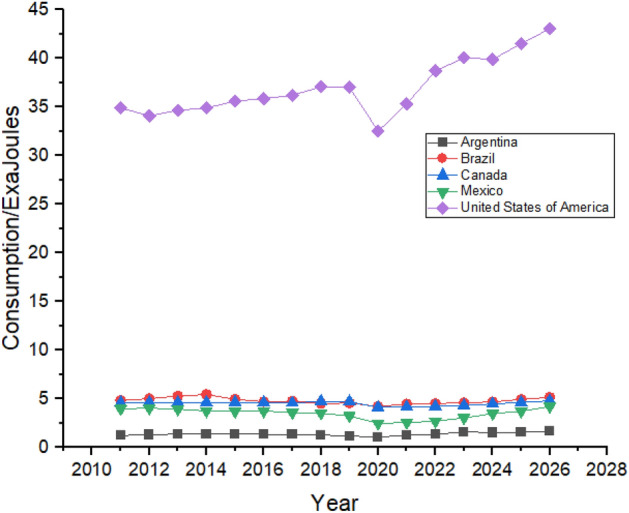
Oil consumption in American Region.

Figure 5 shows the Forecasting of Oil Consumption by G20 countries in continent America. The United States is the biggest consumer of Oil in the American region, followed by Brazil and Canada due to their rapid industrialization^49^. Oil consumption declined in the United States between 2011 and 2014, and it is predicted to be increased from 2022 to 2026. Brazil’s oil consumption showed robust growth between 2011 and 2015 as their oil consumption was 4.83 exajoules in 2011, which increased to 4.94 exajoules in 2015. High consumption of oil forced Brazil to change its energy policy and reduce its oil consumption which they managed to do which can be seen in Fig. 5 which shows that Brazil’s oil consumption reduced to 4.54 exajoules in 2019 However, the forecast period still shows growth as its oil consumption is predicted to be 5.14 exajoules at the end of the forecast period in 2026^50^. Canada is the third-biggest consumer of oil in the American region. However, it still tries to reduce its dependence on oil by controlling oil consumption. Still, during the first three years of the forecast period from 2022 to 2024, oil consumption is predicted to significantly increase in Canada from 4.22 exajoules in 2022 to 4.79 exajoules in 2026. In contrast, the next two years of the forecast period show a decrease in oil consumption.

Oil consumption in Mexico is also predicted to be increased, but it offers still lesser consumption until 2026 than what was being consumed in 2012. Mexico controlled its oil consumption from 2012 to 2019, whereas it is predicted to slightly increase during the forecast period, as shown in Fig. 5. Argentina’s oil consumption shows a weak trend during the forecast period, increasing oil consumption from 1.33 exajoules to 2022 to 1.66 exajoules to 2026.

**Figure 6:**
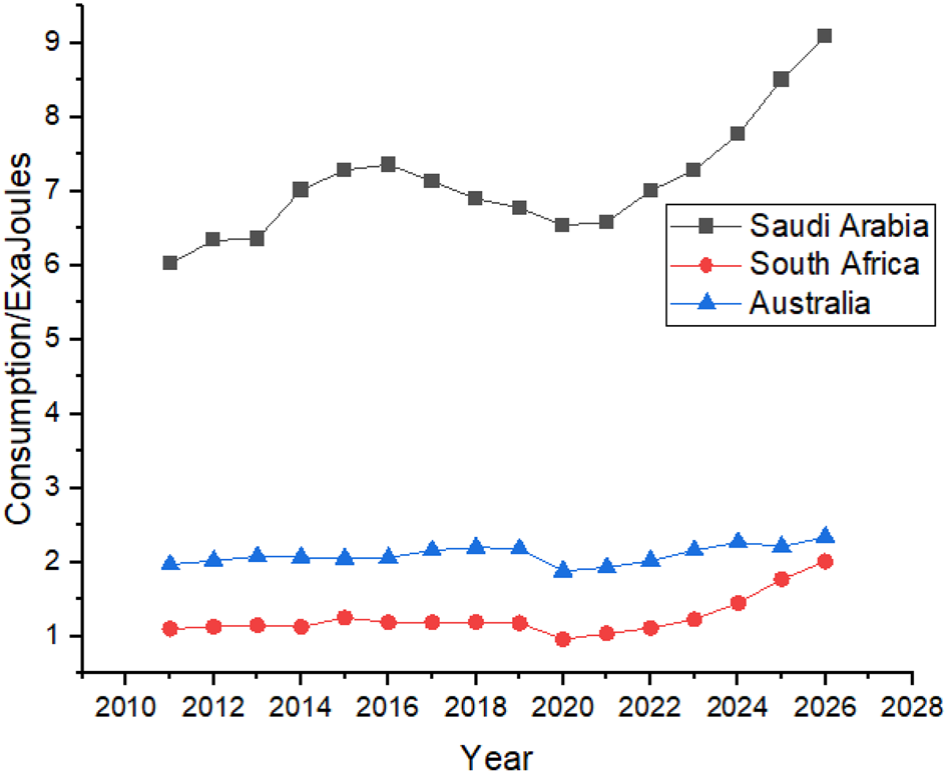
Oil consumption in Australian, African and Middle Eastern Countries.

In Australia, oil consumption increased from 1.97 exajoules in 2011 to 2.18 exajoules in 2019, higher than in South Africa. Oil consumption growth showed a weak trend as it had an oil consumption of 1.11 exajoules in 2011, which then increased to 1.18 exajoules in 2019. During the forecast period, oil consumption is predicted to be increased from 2.02 exajoules to 2.34 and 1.11 exajoules to 2.01 exajoules in Australia and South Africa, respectively, as shown in Fig. 6. Saudi Arabia is the biggest exporter of oil globally and predicted to have a significant and strong increase between 2022 and 2026 as far as oil consumption is concerned, as shown in Fig. 6.

**Figure 7:**
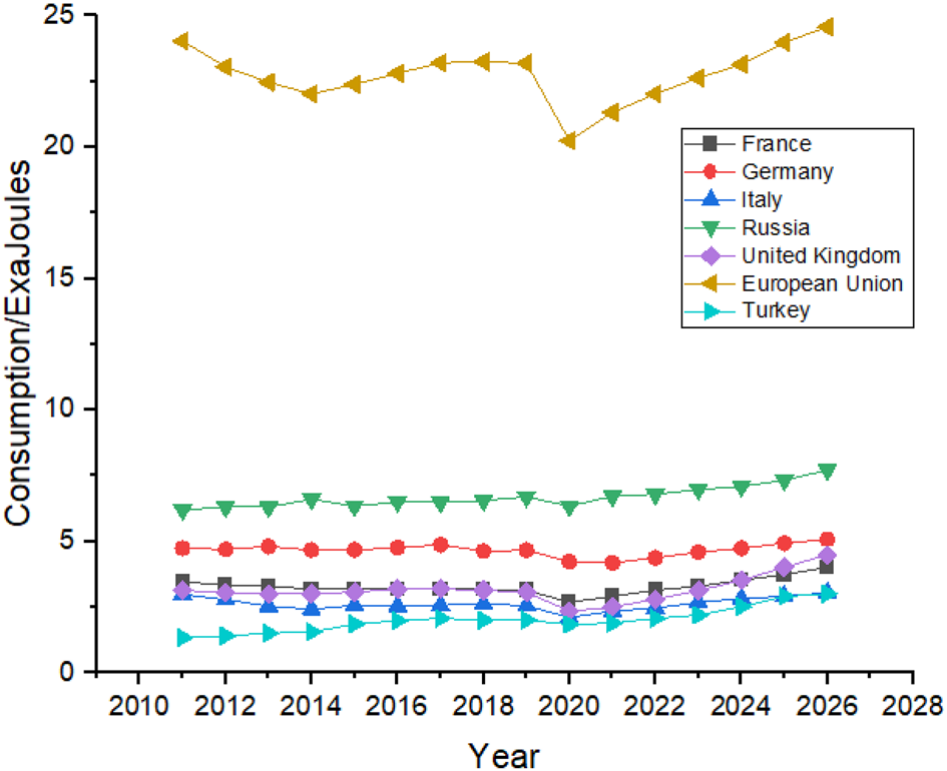
Oil Consumption in European Region.

Figure 7 shows the oil consumption in the European region and in its nearby countries such as Turkey and Russia. European Union and Germany are the biggest oil consumers in Europe, but Germany has the most optimistic stats regarding reducing oil consumption. Germany used 4.73 exajoules of oil in 2011, which decreased to 4.18 exajoules in 2021. Still, it controlled its consumption by relying more on renewable sources due to regional policies, showing that Germany’s oil consumption reduced between 2011 and 2021^51^. During the forecast period, it shows robust growth in its oil consumption which is supposed to be increased from 4.37 exajoules to 5.07 exajoules. France is the only country in the European region that worked on its oil consumption and had managed to consistently reduce it between 2011 and 2021 from 3.45 exajoules to 2.91 exajoules. Still, the forecast period shows linear growth from 3.14 exajoules in 2022 to 4.01 exajoules in 2026. This increase is perhaps due to France’s rise in its oil consumption between 2015 and 2019, as shown in Fig. 7. Italy had reduced its oil consumption between 2.98 exajoules to 2.35 exajoules from 2011 to 2021 by running its industry on renewable energy sources. Still, from 2014 and 2018, its consumption increased from 2.42 exajoules to 2.63 exajoules which slightly declined to 2.35 exajoules in 2021^52^. During the forecast period from 2022 to 2026, the consumption is predicted from 2.46 exajoules in 2022 to 3.04 exajoules in 2026, which is still a weak trend compared to other countries in the region. The United Kingdom had also reduced its oil consumption between 2011 and 2021 from 3.14 exajoules to 2.5 exajoules. Still, after the political situation due to Brexit, it increased to 3.06 exajoules in 2019, which is predicted to increase by healthy trend as Fig. 7 shows that it can grow to 4.49 exajoules in 2026. Turkey is the smallest oil consumers in the region. Turkey shows a very strong growth in its oil consumption as it consumed 1.34 exajoules in 2011 which increased to 1.89 exajoules in 2021 and is predicted to be increased to 3.01 exajoules in 2026. European Union’s oil consumption is predicted to show a weaker trend as its consumption is increase from 24.03 exajoules to 24.57 exajoules between 2011 and 2026.

**Figure 8:**
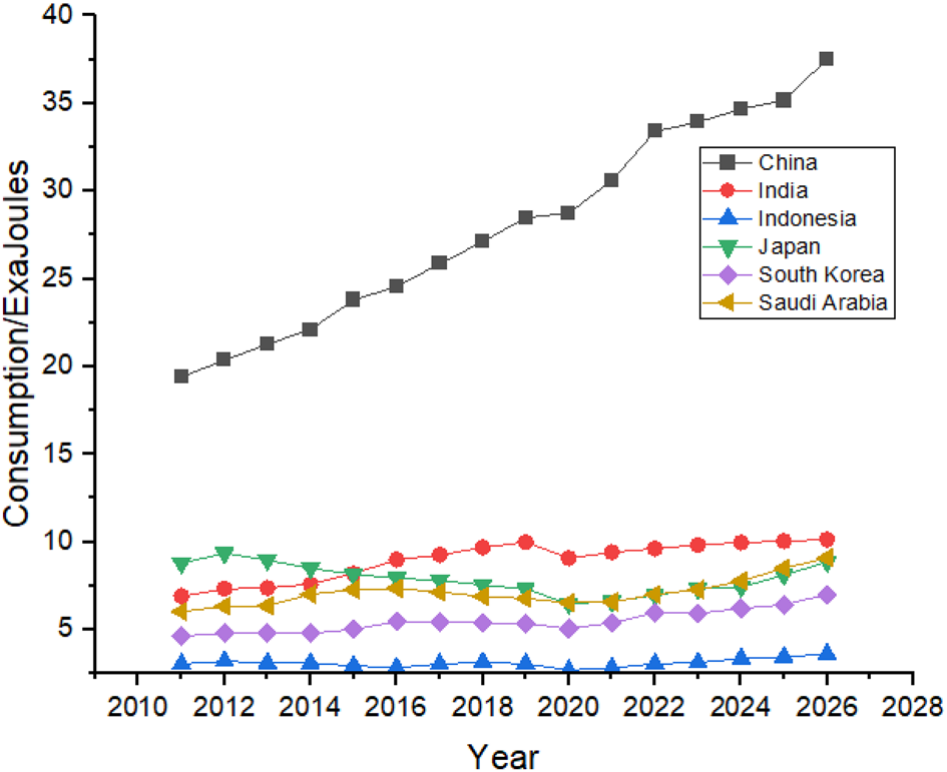
Oil consumption in Asian Region.

Asia has one of the biggest oil consumers among the G20 countries, with China leading the way. China’s oil consumption is the highest globally, increasing by huge numbers between 2011 and 2021, shown in Fig. 8. China was consuming 19.41 exajoules of oil in 2011, which increased by a massive number in the next 10 years, and in 2021 China’s total consumption of oil was 30.6 exajoules. China’s rapid industrialization and urbanization is the main reason behind the massive increase in its oil consumption^53^. During the forecast period, its consumption is predicted to be increased from 30.6 exajoules to 37.52 exajoules. Japan and India follow China in oil consumption in the region. Still, Japan had managed to reduce its oil consumption from 8.78 exajoules in 2011 to 6.61 exajoules in 2021, but in the forecast period, it shows just a slight increase in its oil consumption.

On the contrary, India’s oil consumption has been increasing throughout the studied period as it consumed 6.91 exajoules in 2011, which is supposed to jump to 9.41 exajoules by 2021. Indonesia is the minor oil consumer in Asia, and its consumption is predicted to increase slightly between 2022 and 2026. On the other hand, South Korea has managed to reduce its dependence on oil and is expected to consistently follow the trend between 2022 and 2026.

### Nuclear energy

**Figure 9:**
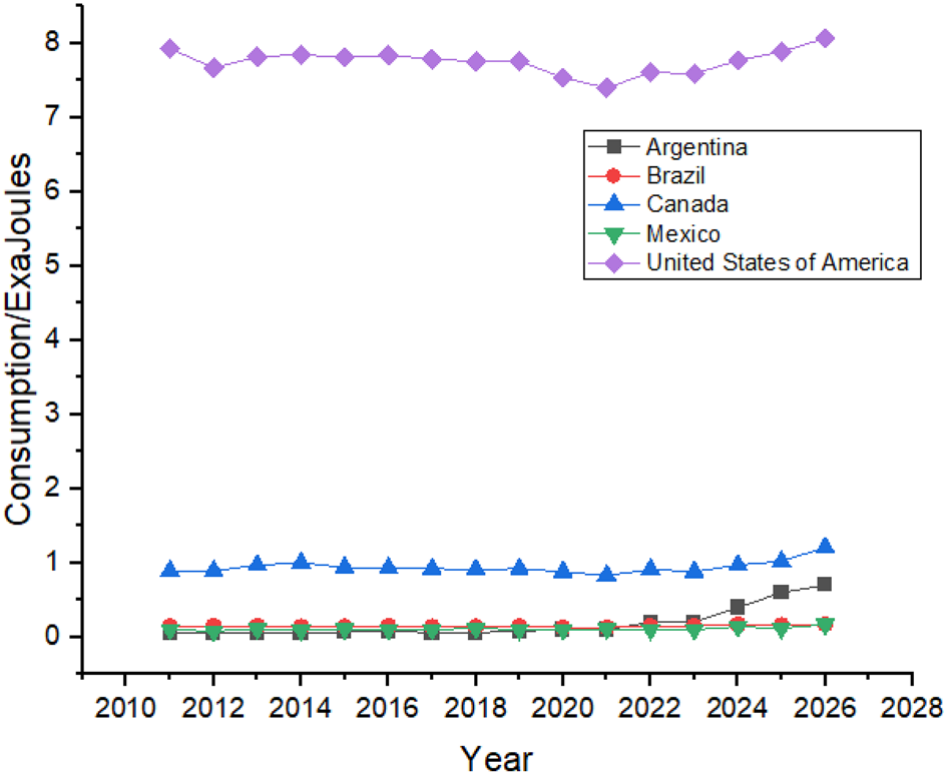
Nuclear energy consumption in American Region.

Figure 9 shows the Forecasting of Nuclear Energy Consumption by in G20 countries of continent America. The United States lead the way in nuclear energy consumption in the American region by consuming 7.93 exajoules in 2011 and is predicted to maintain its consumption during the forecast period. Canada and Brazil are the second and third most significant consumers of nuclear energy in the region, respectively. Brazil’s nuclear energy consumption is predicted to rise during the forecast period, as shown in Fig. 9. In contrast, Canada is expected to consume 1.21 exajoules of atomic energy in 2026 compared to 2021 when they used only 0.83 exajoules. Argentina and Mexico have minor contributions in nuclear energy consumption. In 2021 only 0.1 exajoules and 0.2 exajoules nuclear energy is used by Mexico and Argentina, respectively, which is not predicted to show any substantial growth in the next 5 years according to Fig. 9, which shows the Nuclear Energy Consumption in American Region.

**Figure 10:**
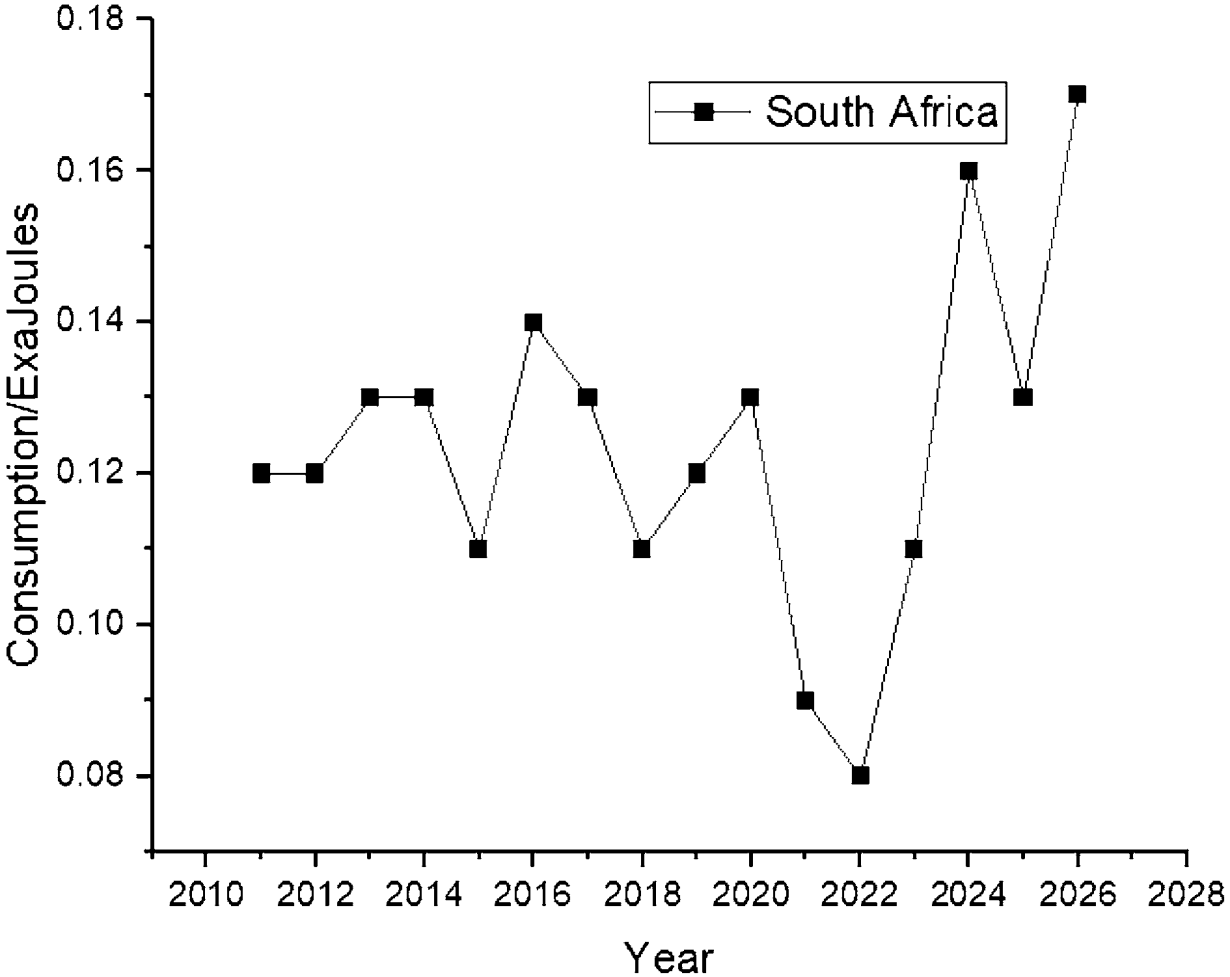
Nuclear energy consumption in South Africa.

Figure 10 shows that Australia’s nuclear energy data is not available, while South Africa’s nuclear energy consumption shows a rising trend during the forecast period between 2022 and 2026, as shown in Fig. 10.

**Figure 11:**
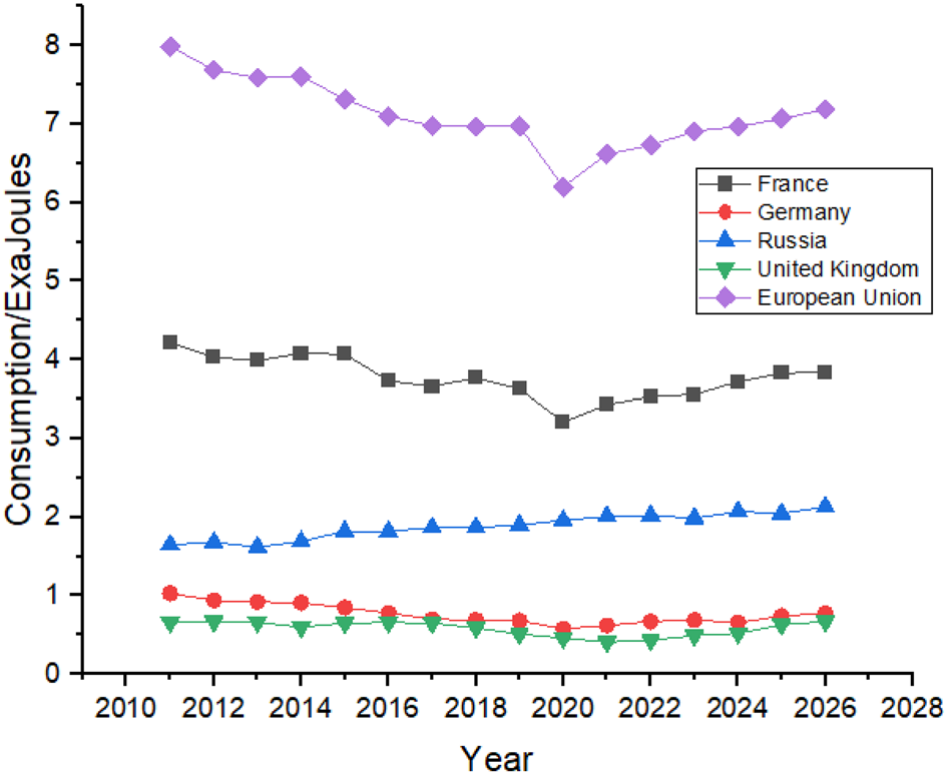
Nuclear energy consumption in European Region.

As shown in Fig. 11, France had reduced its consumption after 2014 and is expected to show a slight increase in its consumption during the forecast period. Russia is expected to have very slim growth over the next 5 years. Russia is also expected to significantly increase its nuclear energy consumption during the forecast period whereas Germany managed to reduce its nuclear energy consumption between 2011 and 2021 by almost half, as shown in Fig. 11, and are predicted to follow the same trend until the end of 2026. The United Kingdom has the lowest nuclear energy consumption in Europe and is expected to increase the consumption to 0.67 exajoules in 2026 from 0.41 exajoules in 2021.

**Figure 12:**
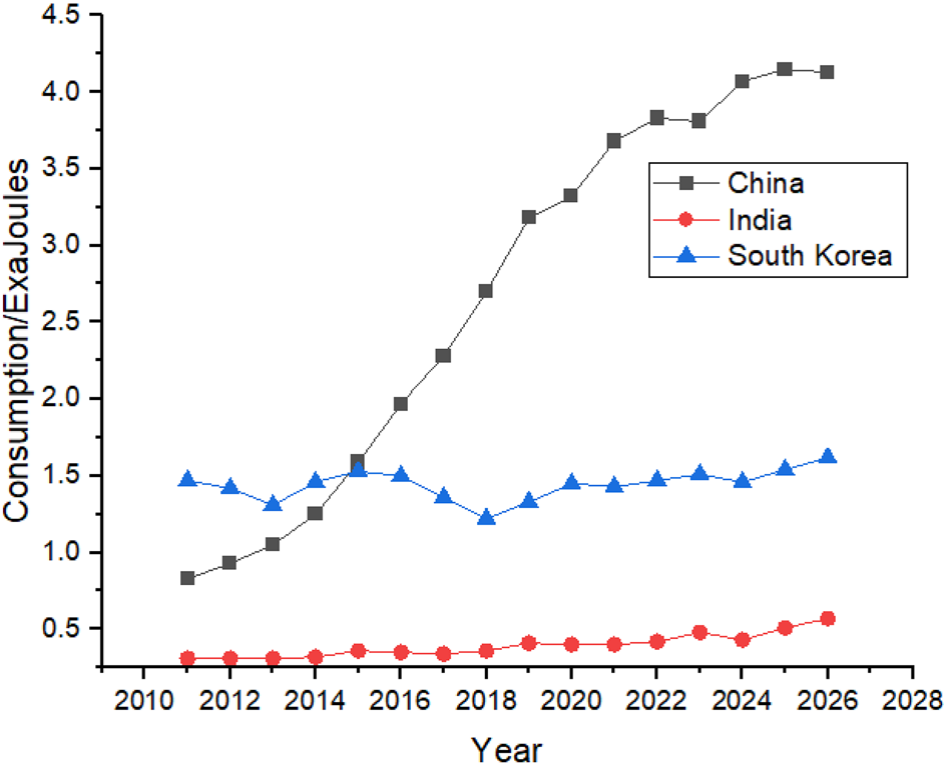
Nuclear energy consumption in Asian Region.

As shown in Fig. 12, China increased its nuclear energy consumption between 2011 and 2021. China’s nuclear energy consumption may increase from 3.68 exajoules in 2021 to 4.13 exajoules in 2026, whereas India’s consumption may increase from 0.4 exajoules in 2021 to 0.57 exajoules in 2026.

### Hydroelectricity

**Figure 13:**
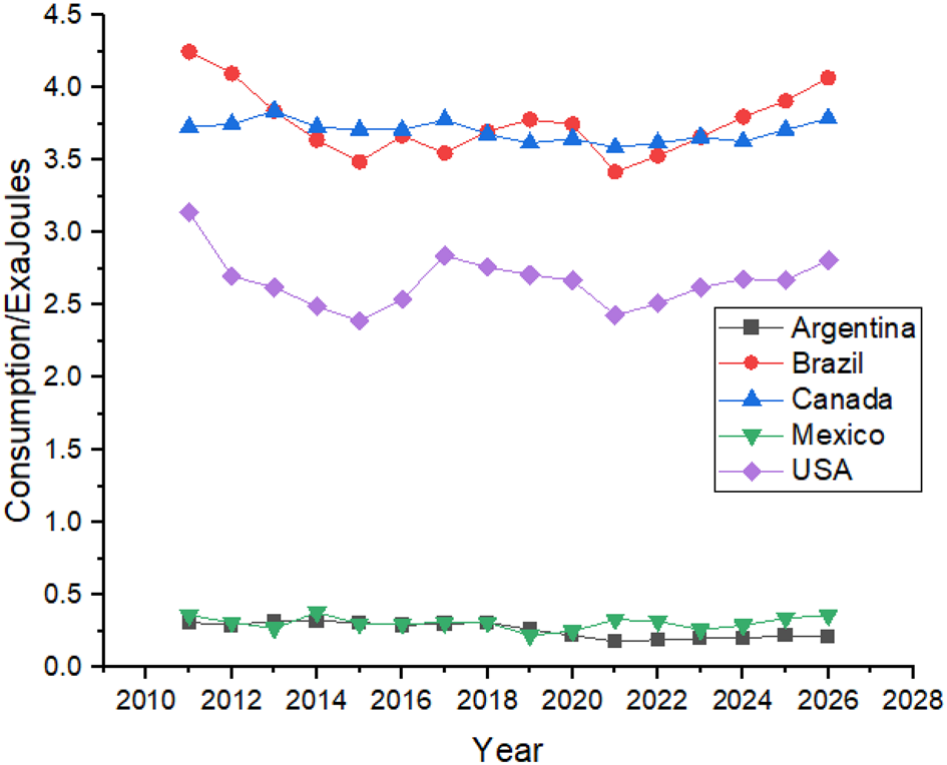
Hydroelectricity consumption in American Region.

Figure 13 shows the Forecasting of Hydroelectricity Consumption by G20 Countries of continent America. Hydroelectricity consumption is not as typical as other non-renewable sources^54^. Canada is the biggest consumer of hydroelectricity in the region due to its rapid demand for dams and other water reserve plants^55^. Its consumption is predicted to increase from 3.62 exajoules in 2021 to 3.79 exajoules in 2026. Brazil and United States are the second and third biggest consumers of hydroelectricity in the studies region, respectively. Both countries show very strong growth in hydroelectricity consumption between 2021 and 2026, as shown in Fig. 13. Mexico and Argentina do not contribute much to hydroelectricity consumption, and they are not predicted to significantly contribute to hydroelectricity consumption at the end of the forecast period.

**Figure 14:**
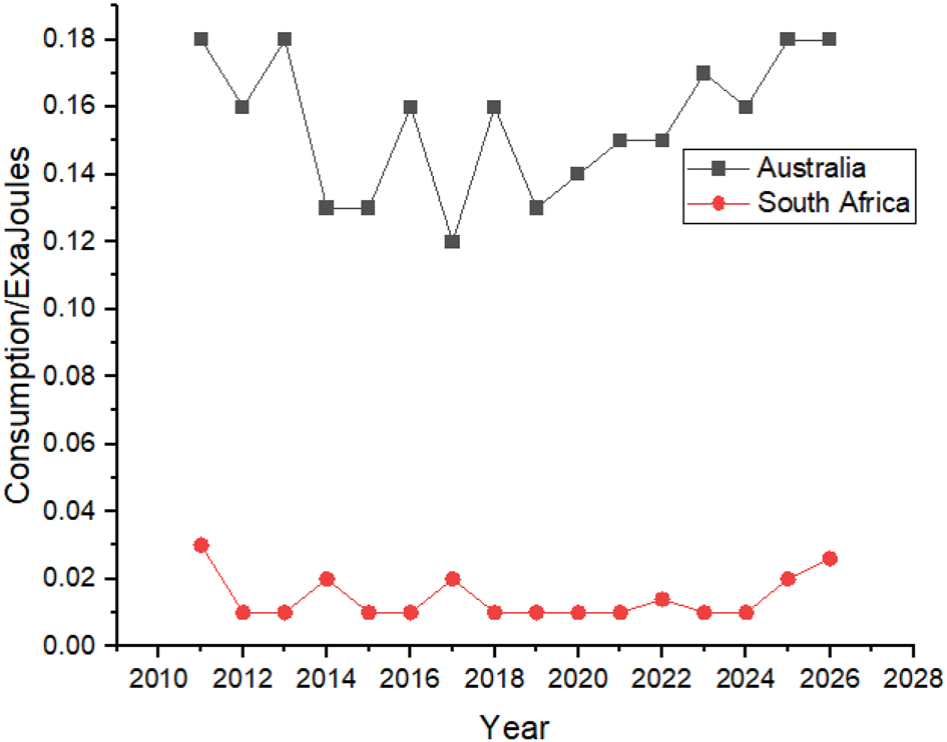
Hydroelectricity consumption in Australia and South Africa.

Hydroelectricity consumption in Australia and South Africa is predicted to increase between 2022 and 2026 as the consumption in Australia is expected to grow from 0.15 exajoules to 0.18 exajoules, whereas in South Africa, from 0.014 exajoules in 2022 to 0.026 exajoules in 2026. Still, in the last 2 years of the forecast period, the hydroelectricity consumption in Australia is predicted to have a similar value as 2011, shown in Fig. 14.

**Figure 15:**
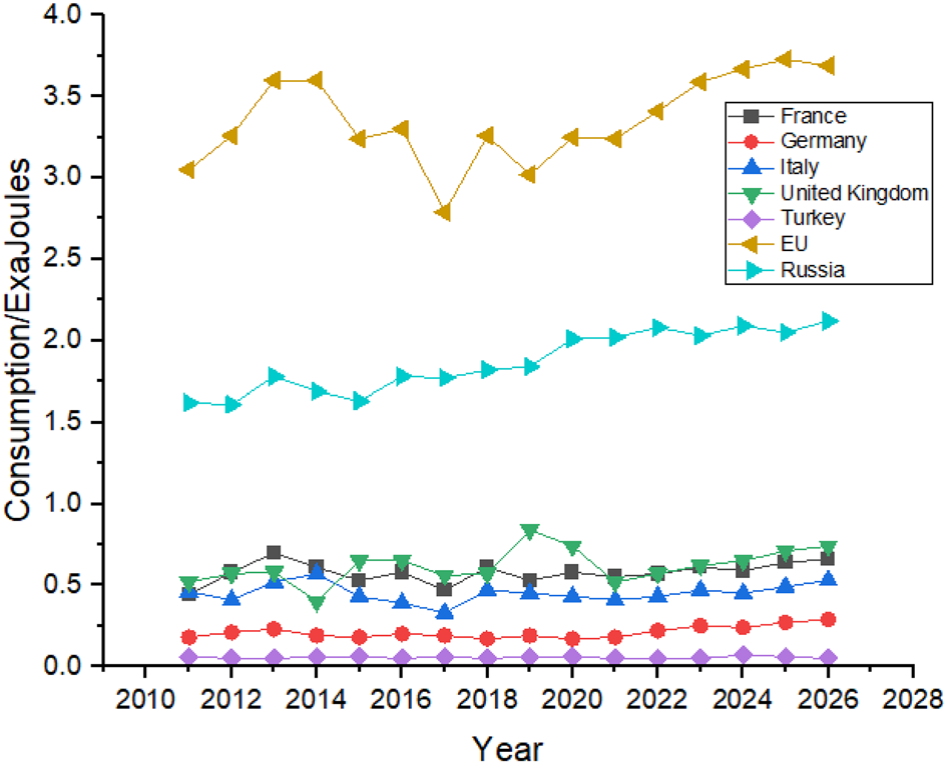
Hydroelectricity consumption in European Region.

As shown in Fig. 15, the European union consumes more hydroelectricity than any other country. During the forecast period, consumption in European Union is expected to increase from 3.41 exajoules in 2022 to 3.69 exajoules in 2026. Russia is the second biggest consumer among the nearby counties of Europe as its consumption increased 1.62 exajoules in 2011 to 2.02 exajoules in 2022 and may further increase to 2.12 exajoules in 2026. Other countries like Italy, Turkey, and Germany show an increase in hydroelectricity consumption between the studied period. The United Kingdom is predicted to have an almost similar trend with no significant increase in its hydroelectricity consumption but may show a slight increase in its hydroelectricity consumption.

**Figure 16:**
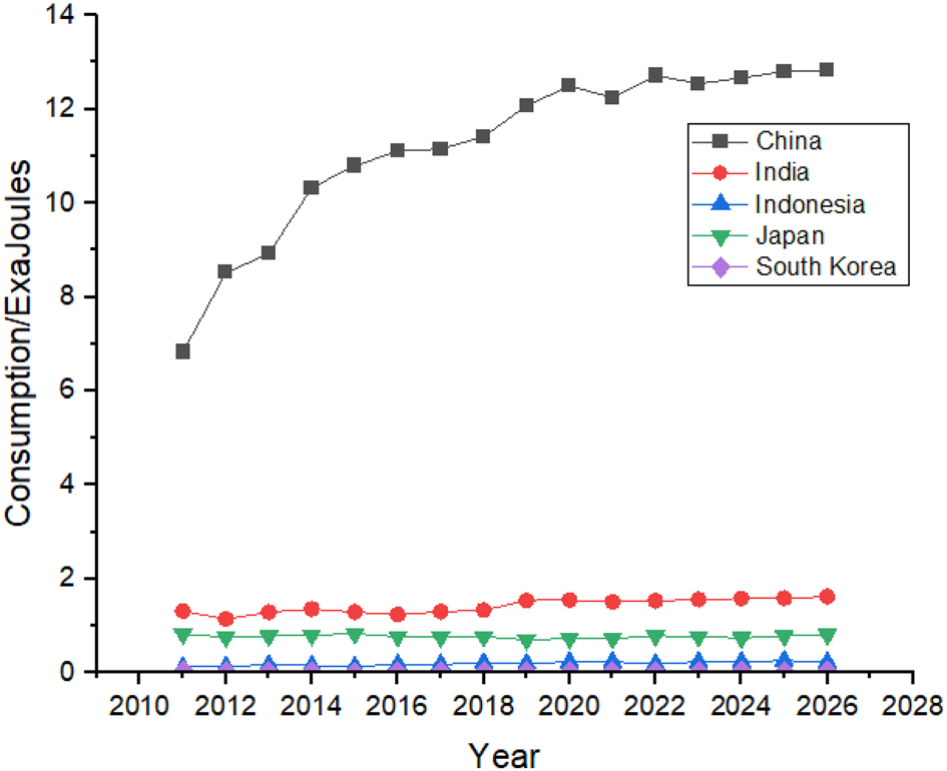
Hydroelectricity consumption in Asian Region.

China consumes hydroelectricity almost equal to all the other countries in Asia. China’s hydroelectricity consumption is expected to keep increasing until 2026. China was consuming 6.83 exajoules in 2011 and is expected to reach the consumption of 12.83 exajoules by 2026. India and Japan are the second and third biggest consumers of hydroelectricity, respectively in the studied region. They are expected to maintain their positions with a minor increase in their hydroelectricity consumption, as shown in Fig. 16. Indonesia is also expected to increase its consumption during 2011 to 2026 whereas South Korea is expected to hold its position throughout the studied period.

### Natural gas

**Figure 17:**
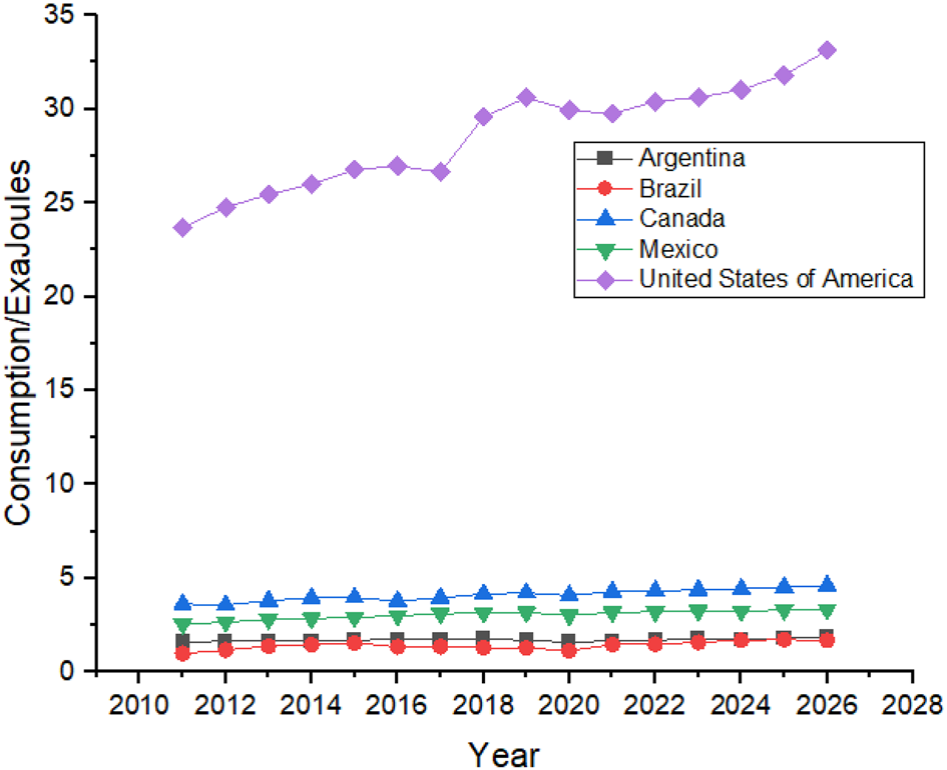
Natural gas consumption in American Region.

Figure 17 shows the forecasting of Natural Gas Consumption by G20 Countries in continent America. There is a massive gap between the countries regarding natural gas consumption^56^. The United States consumes the highest amount of natural gas in the American region. United States natural gas consumption had increased from 23.7 exajoules in 2011 to 29.76 exajoules in 2021, which is predicted to show the same linear growth by 2026 with a consumption of 33.14 exajoules. Canada and Mexico are second and third-biggest natural gas consumers, respectively with rapid growth in their consumption between 2011 and 2021. Canada’s rapid growth in natural gas consumption is caused by the increase in its population, which is considered to remain the same by the end of the forecast period in 2026^57^. Brazil and Argentina are predicted to show minor increase in their natural gas consumption until 2026^58^. Figure 17 shows the Natural Gas Consumption in American Region. It can be observed that natural gas consumption in all the countries dropped down between 2019 and 2021 which may have caused due to COVID-19 as industry had to be shut down for few months as a safety measure.

**Figure 18:**
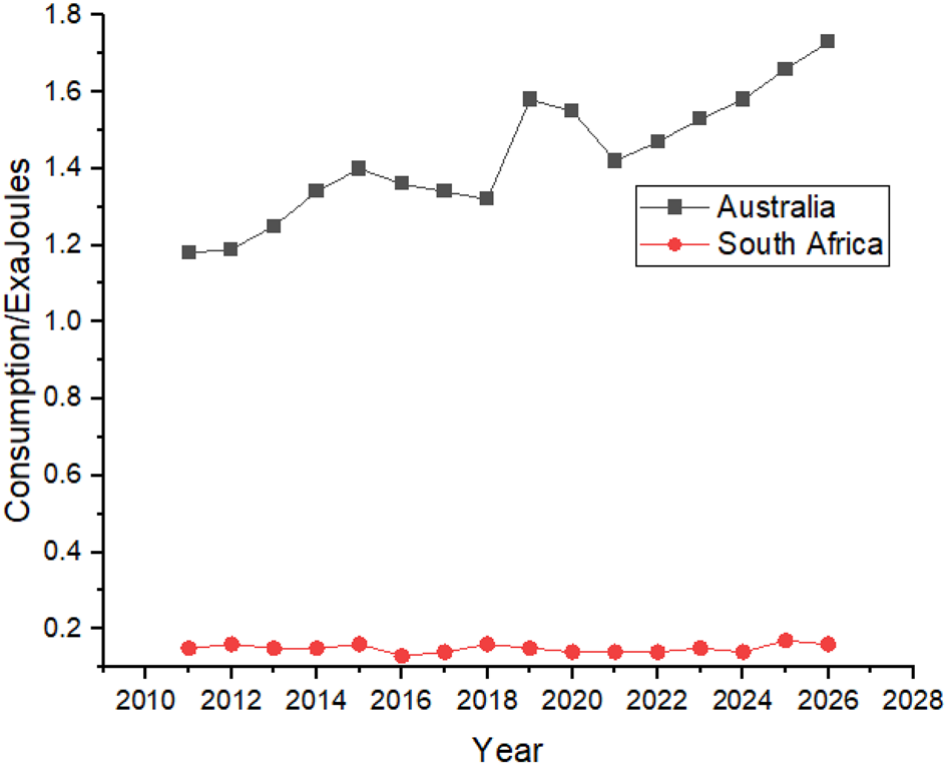
Natural gas consumption in Australia and South Africa.

Australia’s natural gas consumption had shown growth between 2011 and 2026 as it rose to 1.18 exajoules from 1.73 exajoules which is shown in Fig. 18. In contrast, consumption in South Africa has almost similar numbers between the same period, as shown in Fig. 18. A middle eastern country like Saudi Arabia also consumes a high amount of natural gas as they consumed 4.22 exajoules in 2021. It is predicted that the consumption can reach 4.73 exajoules by the end of 2026.

**Figure 19:**
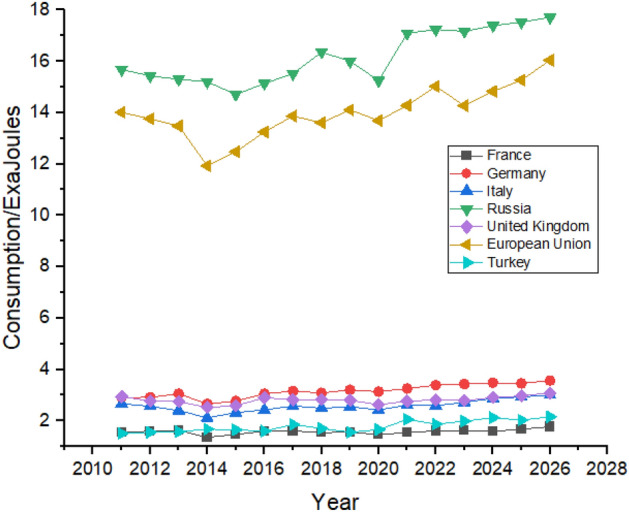
Natural gas consumption in European Region.

Russia led in gas consumption in the region with consumption of 15.68 exajoules in 2011 to 17.09 exajoules in 2021 and is predicted to maintain its position with slight increase in its consumption until the end of 2026. As mentioned earlier, natural gas significantly impacts countries’ progress. Figure 19 shows that in Europe, all the countries are expected to increase their natural gas consumption, with the European Union leading the way from 14.01 exajoules in 2011 to 16.04 exajoules in 2026. In contrast, France and Turkey are the minor consumers of natural gas in the region and are expected to maintain their position until 2026.

**Figure 20:**
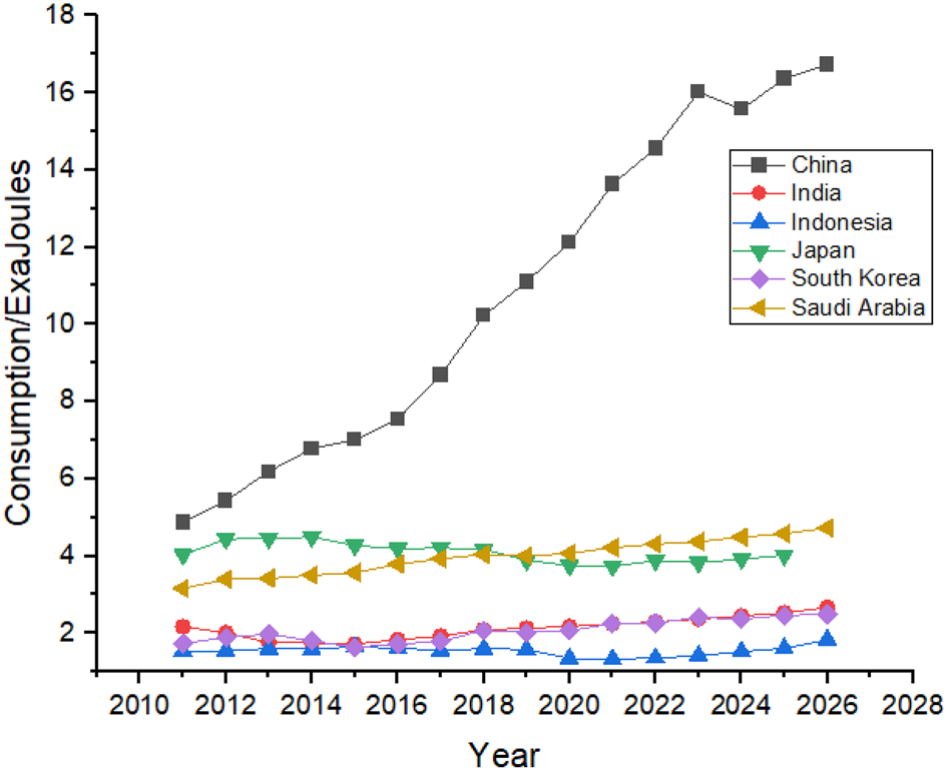
Natural gas consumption in Asian Region.

Rapid growth in natural gas consumption is shown by China, where the consumption was 4.87 exajoules in 2011, and it jumped to 13.63 exajoules in 2021, which is forecasted to reach 16.73 exajoules at the end of 2026, which can be seen in Fig. 20. Other countries like Japan, India, Indonesia, and South Korea also have significant contributions to natural gas consumption. They are expected to remain in the picture with almost same contribution until the end of 2026, with substantial consumption shown by Japan and India.

### Coal

**Figure 21:**
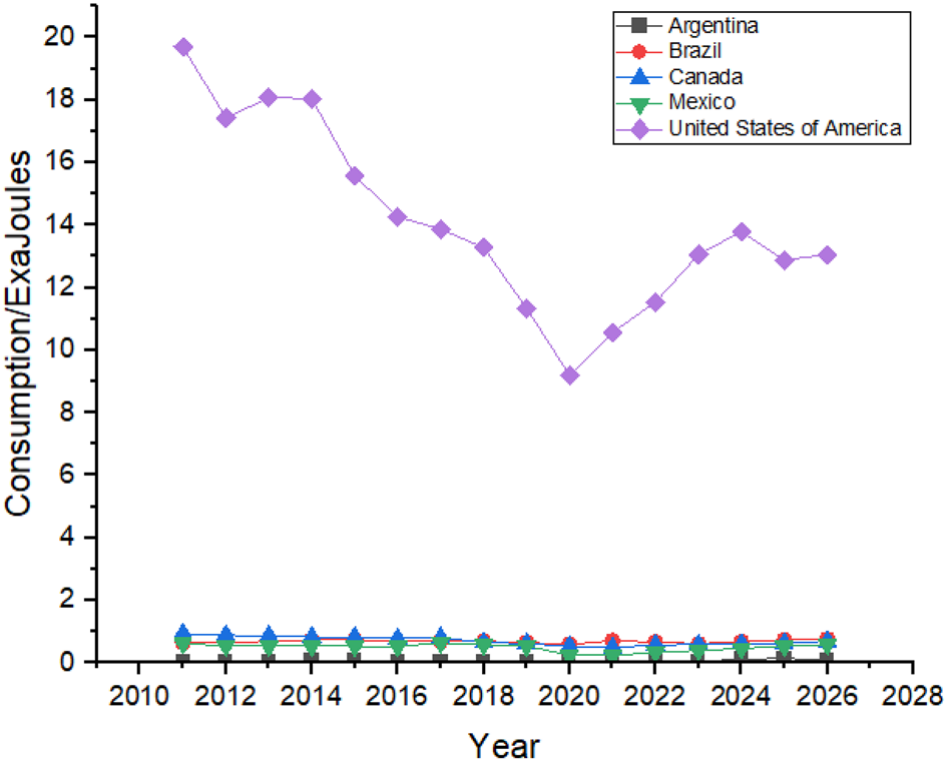
Coal consumption in American Region.

Figure 21 shows the Forecasting of Coal Consumption by G20 Countries in continent America. Coal consumption keeps falling due to it being replaced by natural gas and renewables; hence, coal’s contribution in the energy mix fell to 27%, reaching its lowest level in the last 18 years^59^. Like natural gas, the United States coal is the biggest consumer of coal in the American region. Still, it had shown a decline in coal consumption between 2011 and 2021 as consumption fell from 19.7 exajoules in 2011 to 10.57 exajoules in 2021, which is predicted to follow the same trend during the forecast period with consumption maintaining its position of 2011, to 13.06 exajoules at the end of 2026 according to Fig. 21. Argentina and Brazil are the only countries in the American region that show minor fluctuation in coal consumption, which remains the same in the next five years, as shown in Fig. 21. Canada and Mexico declined their coal consumption between 2011 and 2021, but Mexico decreased its consumption to half between 2011 and 2021.

**Figure 22:**
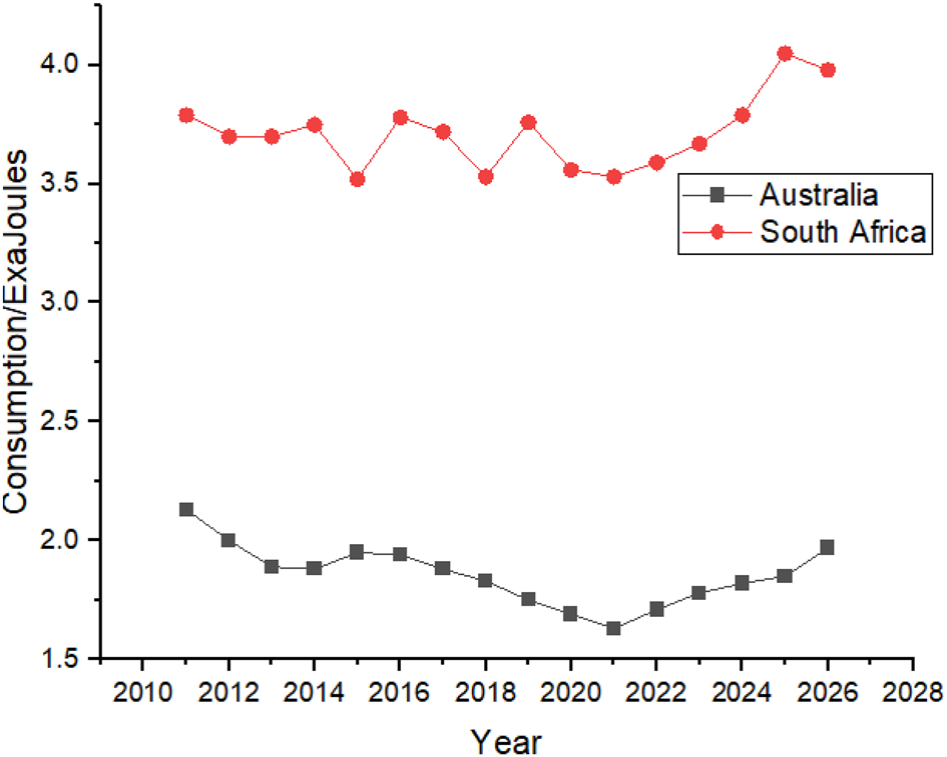
Coal consumption in Australia and South Africa.

Australia’s coal consumption fell from 2.13 exajoules in 2011 to 1.63 exajoules in 2021, and it is predicted to rise its coal consumption slightly to 1.97 exajoules by the end of 2026. On the other hand, South Africa had almost similar coal consumption from 2011 to 2021, which is predicted to remain the same during the forecast period, as shown in Fig. 22.

**Figure 23:**
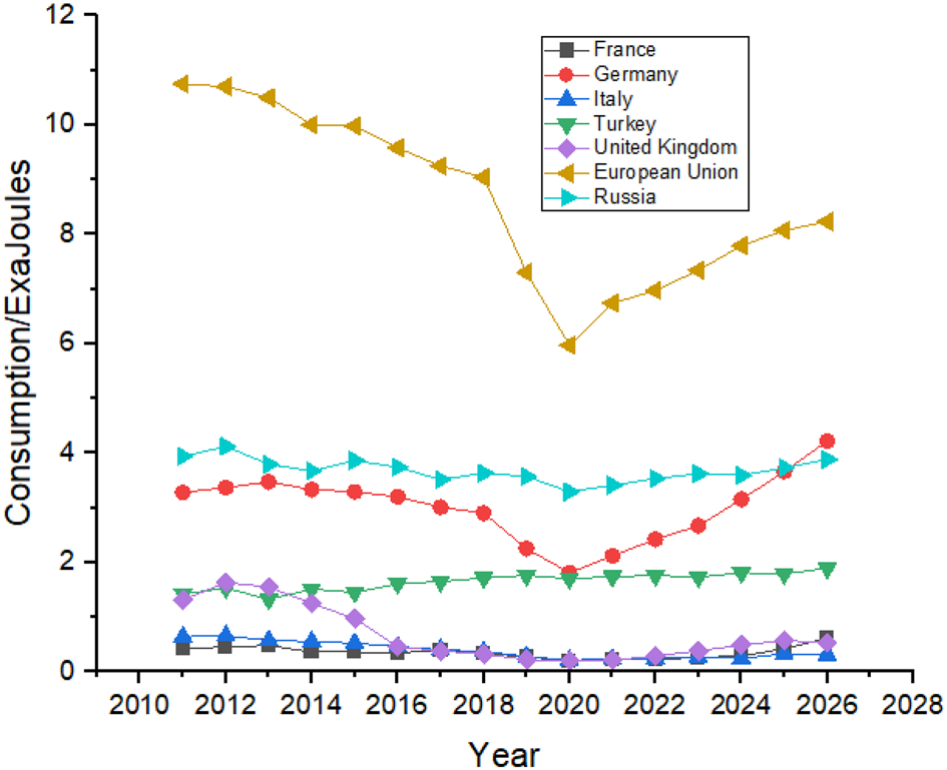
Coal consumption in European Region.

Europe do not have a significant consumer of coal compared to other parts of the world. European Union is the biggest consumer of coal in Europe with a consumption of 10.75 exajoules in 2011 and is expected to consume 8.73 exajoules at the end of 2026 according to the forecasting result shown in Fig. 23. All the countries except Turkey show a decline in their coal consumption between 2011 and 2021. Turkey was consuming 1.42 exajoules of coal in 2011, which reached 1.74 exajoules in 2021 and is predicted to remain the same until the end of the forecast period in 2026, as shown in Fig. 23.

**Figure 24:**
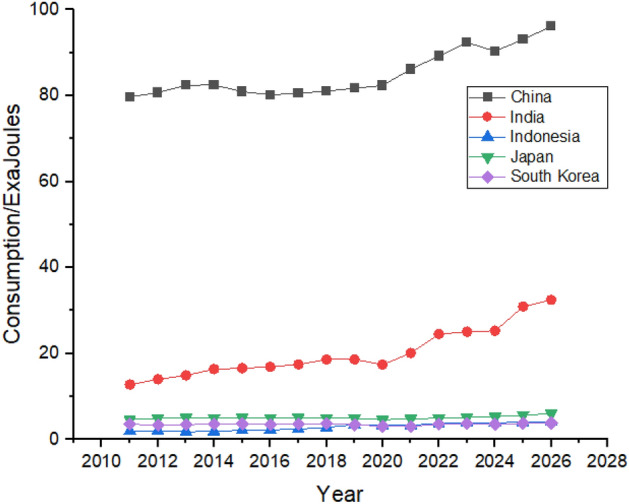
Coal consumption in Asian Region.

China consumes the most amount coal than any other country globally. Coal is the most consumed nonrenewable resource^59^. China’s consumption was 79.71 exajoules in 2011, increasing to 86.17 exajoules in 2021, it is expected to keep fluctuating and may increase during the forecast period and end at a consumption of 96.13 exajoules at the end of 2026, as shown in Fig. 24. Unlike other countries, coal consumption in India and Indonesia is expected to grow significantly during the next 5 years. On the contrary, Japan, and South Korea are expected to decline their coal consumption which can be seen in Fig. 24.

## Conclusion and policy implications

In conclusion, the adjacent accumulation generation operator can be defined by introducing the parameters based on available data. The stability of the studied model is proven by discussing two confirmed cases, and the results were then compared with other forecasting grey models. It is found that more stability was shown exhibited by the disturbance bound of the least squares’ solution. The obtained results predicted that prediction accuracy in many cases could be improved. To analyze the model’s suitability for forecasting, it was interpreted by first predicting the already given data, and the prediction error was then calculated. Hence, DAGM (1,1) can be applied for forecasting as it has an excellent theoretical contribution and shows low error. Furthermore, the practical significance of DAGM (1,1) was proven by applying it to predict the non-renewable energy consumption in G20 countries.

In this paper, the non-renewable energy consumption of G20 countries was analyzed, and the future consumption until 2026 was predicted where DAGM (1,1) was found to be effective. This paper indicates that oil consumption has an increasing trend in all the G20 countries, with the USA leading by China until 2026. Many G20 countries do not make their nuclear energy consumption data available. Based on available data, it can be predicted that China, France, and Russia are expected to consume the most nuclear energy until 2026, with Argentina being the least. Coal is predicted to show a downfall in its consumption by G20 countries except for South Africa and France. Argentina is expected to consume a minuscule amount of coal, with a consumption of 0.09 until 2026. Hydroelectricity is the least consumed form of nonrenewable energy and is predicted to maintain its position until 2026. China, the USA, and European Union are expected to maintain their position as the biggest consumers of hydroelectricity among the G20 countries. Natural gas consumption is predicted to show a massive rise in G20 countries, especially in Russia, China, and the USA, justifiable considering their economic progress. The results can help authorities make the decision more easily as a proven forecasting method is used in this study. It may have policy implications especially related to natural gas and Oil as the obtained data demands reduction in these two non-renewable energy consumption to not only reduce the carbon dioxide emission but also prevent the energy shortfall in the future.

As a new forecasting model, the studied model DAGM (1,1) can be combined with other models to obtain better and optimized results. Considering the prospects of this model, this forecasting grey model can be introduced with time series analysis on large sample data, unlike other forecasting models that are unsuitable for data with larger samples.

## Data Availability

The datasets used and/or analysed during the current study available from the corresponding author on reasonable request.

## Abbreviations

G20: Great 20
MAE: Mean absolute error
RMSE: Root mean square error
MAPE: Mean absolute percentage error
DAGM: Discrete adjacent grey model
EOGM: Exponential optimization grey model
ARX: Autoregressive exogenous
OECD: Organization for Economic Co-operation and Development
